# The family of Deg/HtrA proteases in plants

**DOI:** 10.1186/1471-2229-12-52

**Published:** 2012-04-20

**Authors:** Holger Schuhmann, Pitter F Huesgen, Iwona Adamska

**Affiliations:** 1 Department of Plant Physiology and Biochemistry, University of Konstanz, Universitätsstr. 10, 78457, Konstanz, Germany; 2 School of Agriculture and Food Sciences, University of Queensland, St. Lucia, QLD, 4072, Australia; 3 Centre for Blood Research, University of British Columbia, 2350 Health Sciences Mall, Vancouver, BC, V6T 1Z3, Canada

## Abstract

**Background:**

The Deg/HtrA family of ATP-independent serine endopeptidases is present in nearly all organisms from bacteria to human and vascular plants. In recent years, multiple deg/htrA protease genes were identified in various plant genomes. During genome annotations most proteases were named according to the order of discovery, hence the same names were sometimes given to different types of Deg/HtrA enzymes in different plant species. This can easily lead to false inference of individual protease functions based solely on a shared name. Therefore, the existing names and classification of these proteolytic enzymes does not meet our current needs and a phylogeny-based standardized nomenclature is required.

**Results:**

Using phylogenetic and domain arrangement analysis, we improved the nomenclature of the Deg/HtrA protease family, standardized protease names based on their well-established nomenclature in *Arabidopsis thaliana*, and clarified the evolutionary relationship between orthologous enzymes from various photosynthetic organisms across several divergent systematic groups, including dicots, a monocot, a moss and a green alga. Furthermore, we identified a “core set” of eight proteases shared by all organisms examined here that might provide all the proteolytic potential of Deg/HtrA proteases necessary for a hypothetical plant cell.

**Conclusions:**

In our proposed nomenclature, the evolutionarily closest orthologs have the same protease name, simplifying scientific communication when comparing different plant species and allowing for more reliable inference of protease functions. Further, we proposed that the high number of Deg/HtrA proteases in plants is mainly due to gene duplications unique to the respective organism.

## Background

Proteolysis, the enzyme-mediated hydrolysis of peptide bonds, is a vital process for every organism. It is associated with many intracellular and extracellular events, e.g. the removal of damaged proteins, nutrient uptake, processing of protein precursors, and signaling [1,2]. A huge variety of proteolytic enzymes, utilizing several different catalytic mechanism, mediate these processes. The family of Deg proteases (for degradation of periplasmic proteins) [3], also known as HtrA proteases (for high temperature requirement A) [4], are one important group of these proteolytic enzymes. They are ATP-independent serine endopeptidases found in all domains of life, including Bacteria, Archaea and Eukarya. Deg/HtrA proteases belong to the S1B subfamily of the clan PA according to MEROPS nomenclature [5], which features a catalytic domain of the trypsin type, with His-Asp-Ser as catalytic triad. Most Deg/HtrA family members contain one to four PDZ protein-protein interaction domains [6], but members without PDZ domains have been described in plants [7]‐[9] and animals [8,10]. Deg/HtrA proteases are best studied in *Escherichia coli* and mammals, where three (DegP, DegQ and DegS) or five (HtrA1-4 and Tysnd1) Deg/HtrA paralogs are present, respectively. DegP from *E. coli* is a protein quality control enzyme in the periplasm, acting as a protease and degrading irreversibly damaged proteins, or as a chaperone, thereby assisting with refolding of denaturated proteins [11]. A second *E. coli* protease, DegS, acts in a stress signaling cascade sensing misfolded proteins in the periplasm and transducing the signal to the cytoplasm [12]. Human Deg/HtrA proteases have been shown to play critical roles in severe diseases, such as Alzheimer, age-related macular degeneration and several cancers (reviewed in [13]). Compared to the vast literature on prokaryotic and mammalian Deg/HtrA proteases, relatively little is known about members of this family in plants. Searches in genomic databases revealed 16 genes encoding putative Deg/HtrA proteases in *Arabidopsis thaliana*[14], 15 in *Oryza sativa*[15] and 20 in *Populus trichocarpa*[16]. However, to date only a few Deg/HtrA proteases from *A. thaliana* have been studied in detail. It was experimentally shown that six AtDeg proteases are located in chloroplasts [17]‐[22], one in peroxisomes [8], one in mitochondria [E. Zeiser, C. Huber, P. Huesgen, H. Schuhmann, I. Adamska, unpublished], and one in the nucleus [23]. Two more Deg proteases are predicted to reside in chloroplasts, five in mitochondria (one of them with a possible dual chloroplastidial/mitochondrial localization), and the subcellular location of one protein is uncertain (reviewed [24]). The chloroplast-located Deg/HtrA proteases were reported to be involved in the degradation of damaged photosynthetic proteins, especially the photosystem II (PSII) reaction center D1 protein under light stress conditions (reviewed [24]). Additionally, the thylakoid lumen-located AtDeg1 protease acts as a chaperone, assisting in the assembly of PSII dimers and supercomplexes [25]. Little is known about Deg/HtrA proteases targeted to compartments other than the chloroplast. However, it was demonstrated that the peroxisomal AtDeg15 protease is a processing enzyme, cleaving the N-terminal peroxisomal targeting signal 2 that is present in some nuclear-encoded peroxisomal proteins [7,8]. Based on the evolutionary relationship of the conserved trypsin domain, Deg/HtrA proteases from Archaea, Bacteria and Eukarya cluster into four distinct clades, whereby plants are the only organisms containing proteases from all four clades [7]. The relatively high number of Deg/HtrA proteases and their diversity in plants, together with the observation that some of them localize to the same compartment, have a similar domain arrangements, and comparable sizes [7,14,16], carries a high risk of confusion. This is potentiated by the fact that during genome annotation of vascular plants (e.g. *A. thaliana* and *O. sativa*), Deg/HtrA proteases were numbered according to the order of their discovery, thus giving orthologous proteins different numbers and names depending on the organism. For rice, the situation is even more complex with two independent genome annotation databases for *O. sativa* ssp. *japonica*, i.e. the Rice Annotation Database [26] and the MSU Rice Genome Annotation Project Database [27]. Therefore, one gene might occur in the literature under more than one identifier or name. In the study presented here, we reassessed the number of Deg/HtrA proteases in several photosynthetic eukaryotic model organisms from the *Viridiplantae* line, such as the dicots *A. thaliana* and *P. trichocarpa*, the monocot *O. sativa*, the moss *Physcomitrella patens* and the unicellular green alga *Chlamydomonas reinhardtii*, whose genomes are completely sequenced. Using phylogenetic comparison and domain structure analysis, we propose a unified nomenclature for Deg/HtrA proteases in green plants (including green algae) based on the long-established nomenclature reported for *A. thaliana*[28]. Furthermore, we were able to identify a “core set” of eight Deg/HtrA proteases shared by all organisms examined here and postulate that the high number of Deg/HtrA proteases in plants is mainly due to gene duplications unique to the respective organism.

## Results and discussion

### An inventory of Deg/HtrA proteases

To establish a standardized nomenclature, we reassessed the number of Deg/HtrA proteases in the vascular plants *O. sativa* ssp. *japonica*and *P. trichocarpa*, the moss *P. patens* and the green alga *C. reinhardtii* by searching annotated genome databases for the presence of *deg/htrA* sequences (see Methods for details). The secondary structure of these sequences was analyzed using the HHpred platform [29] in order to confirm the presence of a Deg/HtrA protease domain, thereby excluding false positives from the database searches (data not shown). Additionally, this approach also yielded the domain architecture of con firmed Deg/HtrA proteases, which is included in Tables 1,2,3,4,5.

**Table 1:** **The family of Deg/HtrA proteases in *Arabidopsis thaliana***

**Gene model**^**a**^	**Protein name**^**b**^	**UniProtKB acc. no.**^**c**^	**aa**	**domain arrangement**^**d**^	**Orthologs in other plants (this study)**	**Protein name used in this study**
At3g27925	DEG 1	O22609	439	PD-PDZ	Cre02.g088400 Cre14.g630550 Cre12.g498500 Os05g0568900 Pp1s160_79V6 Pp1s198_100V6 POPTR_0001s34960	AtDeg1
At2g47940	DEG 2	O82261	607	PD-PDZ-PDZ	Cre19.g752200 Os05g0147500 Pp1s8_140V6 POPTR_0014s12970 POPTR_0020s00220	AtDeg2
At1g65630	DEG 3	Q9SHZ1	559	PD-PDZ-PDZ	Deg 10 Subgroup	AtDeg3
At1g65640	DEG 4	Q9SHZ0	518	PD-PDZ-PDZ	Deg10 Subgroup	AtDeg4
At4g18370	DEG 5	Q9SEL7	323	PD	Cre02.g110600 Os12g0616600 Pp1s63_95V6 POPTR_0011s02330	AtDeg5
At1g51150	DEG 6	Q9C691	219	PD_ia_	n.a.	AtDeg6
At3g03380	DEG 7	Q8RY22	1097	PD-PDZ-PDZ- PD_ia_-PDZ-PDZ	Cre03.g180650 Os02g0712000 Pp1s237_5V6 Pp1s21_327V6 POPTR_0017s03050 POPTR_0004s08740 POPTR_0004s08720	AtDeg7
At5g39830	DEG 8	Q9LU10	448	PD-PDZ	Cre01.g028350 Os04g0459900 Pp1s31_50V6 POPTR_0004s13440	AtDeg8
At5g40200	DEG9	Q9FL12	592	PD-PDZ-PDZ	Cre19.g752200 Os02g0742500 Os06g0234100 Pp1s176_87V6 Pp1s1_203V6 POPTR_0015s08440 POPTR_0004s13440	AtDeg9
At5g36950	DEG10	Q9FIV6	586	PD-PDZ-PDZ	Cre14.g617600 Cre01.g013300 Os05g0417100 Pp1s55_7V5.1 POPTR_0008s07940	AtDeg10
At3g16540	DEG11	Q9LK71	555	PD-PDZ-PDZ	Deg10 Subgroup	AtDeg11
At3g16550	DEG12	Q9LK70	499	PD-PDZ-PDZ	Deg10 Subgroup	AtDeg12
At5g40560	DEG13	Q9FM41	486	PD-PDZ-PDZ	Deg10 Subgroup	AtDeg13
At5g27660	DEG14	Q3E6S8	429	PD-PDZ	Os11g0246600 Pp1s180_15V6 POPTR_0013s01900	AtDeg14
At1g28320	DEG15	Q8VZD4	709	NT-PD	Cre12.g548200 Os05g0497700 Pp1s196_28V6 POPTR_0004s04650 POPTR_0011s05510	AtDeg15
At5g54745	DEG16	Q3E8B4	198	PD_ia_	n.a.	AtDeg16

^a^ According to TAIR database. ^b^ According to [14]. ^c^ If more than one protein entry was present, the different versions were analyzed by the HHPred platform (http://toolkit.tuebingen.mpg.de/hhpred/), and the one with intact protease domain and (if present) PDZ domain(s) was considered here. Sequences used in this study are supplied as Supplementary material (Additional file 1). ^d^ According to the HHPred platform. Abbreviations: aa, amino acids; n.a., not available; NT, elongated N-terminus; PD, potentially active protease domain; PD_ia_, inactive protease domain (i.e. at least one residue of the catalytic triad is mutated or missing); PDZ, PDZ domain.

**Table 2:** **The family of Deg/HtrA proteases in *Populus trichocarpa***

**Gene model**^**a**^	**Protein name**^**b**^	**UniProtKB acc. no.**^**c**^	**aa**	**domain arrangement**^**d**^	**Orthologs in other plants (this study)**	**Proposed protein name**
POPTR_0001s34960 Pt706718	PtDeg1	A9PI52	429	PD-PDZ	At3g27925 Cre02.g088400 Cre14.g630550 Cre12.g498500 Os05g0568900 Pp1s160_79V6 Pp1s198_100V6	**PtDeg1**
POPTR_0014s12970 Pt572750	PtDeg2.1	B9I9X1	592	PD-PDZ-PDZ	At2g47940 Cre19.g752200 Os05g0147500 Pp1s8_140V6	**PtDeg2.1**
POPTR_0020s00220 Pt775566	PtDeg2.2	B9IBU0	624	PD-PDZ-PDZ	At2g47940 Cre19.g752200 Os05g0147500 Pp1s8_140V6	**PtDeg2.2**
POPTR_0011s02330 Pt771291	PtDeg5.1	B9HYW4	316	PD	At4g18370 Cre02.g110600 Os12g0616600 Pp1s63_95V6	**PtDeg5**
POPTR_0017s03050 Pt816849	PtDeg7.1	B9GV35	1128	PD-PDZ-PDZ- PD_ia_-PDZ-PDZ	At3g03380 Cre03.g180650 Os02g0712000 Pp1s237_5V6 Pp1s21_327V6	**PtDeg7.1**
POPTR_0004s08740 Pt555951	PtDeg7.2	B9H390	1080	PD-PDZ-PDZ- PD_ia_-PDZ-PDZ	At3g03380 Cre03.g180650 Os02g0712000 Pp1s237_5V6 Pp1s21_327V6	**PtDeg7.2**
POPTR_0004s08720 Pt714140	PtDeg7.3	B9H391	1117	PD-PDZ-PDZ- PD_ia_-PDZ-PDZ	At3g03380 Cre03.g180650 Os02g0712000 Pp1s237_5V6 Pp1s21_327V6	**PtDeg7.3**
POPTR_0004s13440 Pt199267	PtDeg8	B9H3X7	465	PD-PDZ	At5g39830 Cre01.g028350 Os04g0459900 Pp1s31_50V6	**PtDeg8**
POPTR_0015s08440 Pt251989	PtDeg9.1	B9IEN8	556	PD-PDZ-PDZ	At5g40200 Cre19.g752200 Os02g0742500 Os06g0234100 Pp1s176_87V6 Pp1s1_203V6	**PtDeg9.1**
POPTR_0012s07930 Pt728836/Pt823359	PtDeg9.2	B9I375	559	PD-PDZ-PDZ	At5g40200 Cre19.g752200 Os02g0742500 Os06g0234100 Pp1s176_87V6 Pp1s1_203V6	**PtDeg9.2**
POPTR_0008s07940		B9HI10	587	PD-PDZ-PDZ	At5g36950 Cre01.g013300 Cre14.g617600 Os05g0417100 Pp1s55_7V5.1	**PtDeg10**
POPTR_0013s01900 Pt662713/Pt662714	PtDeg14.1 PtDeg14.2	B9I7J6 (partial)	422	PD-PDZ	At5g27660 Os11g0246600 Pp1s180_15V6	**PtDeg14**
POPTR_0004s04650 Pt555773	PtDeg15.1	B9H2S3	752	NT-PD	At1g28320 Cre12.g548200 Os05g0497700 Pp1s196_28V6	**PtDeg15.1**
POPTR_0011s05510 Pt266544	PtDeg15.2	B9N3H9	729	NT-PD	At1g28320 Cre12.g548200 Os05g0497700 Pp1s196_28V6	**PtDeg15.2**
POPTR_0018s04140 Pt787034	PtDeg17.1	B9NA38	356	PD_ia_-PDZ	n.a.	**PtDeg17.1**
POPTR_0394s00220 Pt586371	PtDeg17.2	B9NA39 (fragment)	298	PD_ia_-PDZ	n.a.	**PtDeg17.2**
POPTR_0018s04150 Pt577788	PtDeg17.3	B9INA2	364	PD_ia_-PDZ	n.a.	**PtDeg17.3**

^a^ First model identifier is from Phytozome v7.0 (http://www.phytozome.net), the second identifier is the corresponding identifier according to [16]. Discrepancies between the suggested gene model and the UniprotKB entry were solved by analyzing the EST data (if present) and analysis of the genomic sequence for the presence of ORFs yielding aa sequences similar to ortholog or paralog proteins, with respect to potential splicing sites.^b^ According to [16]^c^ If more than one protein entry was present, the different versions were analyzed by the HHPred platform (http://toolkit.tuebingen.mpg.de/hhpred/), and the one with intact protease domain and (if present) PDZ domain(s) was considered here. Sequences used in this study are supplied as Supplementary material (Additional file 1). ^d^ According to the HHPred platform. Abbreviations: aa, amino acids; n.a., not available; NT, elongated N-terminus; PD, potentially active protease domain; PD_(1/2)_, truncated protease domain, probably proteolytically inactive; PD_ia_, inactive protease domain (i.e. at least one residue of the catalytic triad is mutated, or protease domain is incomplete); PDZ, PDZ domain.

**Table 3:** **The family of Deg/HtrA proteases in *Oryza sativa***

**Gene model**^**a**^	**Previouis protein name**^**b**^	**UniProtKB acc. no.**^**c**^	**aa**	**Domain arrangement**^**d**^	**Orthologs in other plants (this study)**	**Proposed protein name**
Os01g0278600 LOC_Os01g17070	Os01g0278600 OsDegP1	Q5NBK7	470	PD_ia_-PDZ	n.a.	**OsDeg-like 1**
Os02g0712000 LOC_Os02g48180	Os02g0712000 OsDegP2	Q6ZIR2/B9F2C1	1092^e^	PD-PDZ-PDZ- PD_ia_-PDZ-PDZ	At3g03380 Cre03.g180650 Pp1s237_5V6 Pp1s21_327V6 POPTR_0017s03050 POPTR_0004s08740 POPTR_0004s08720	**OsDeg7**
Os02g0742500 LOC_Os02g50880	Os02g0742500 OsDegP3	Q6Z806	567	PD-PDZ-PDZ	At5g40200 Cre19.g752200 Pp1s176_87V6 Pp1s1_203V6 POPTR_0015s08440 POPTR_0004s13440	**OsDeg9.1**
-LOC_Os03g62900	-OsDegP4	Q84SQ1	299	PD	n.a. – not a Deg?	**OsDeg-like 6**
Os04g0459900 LOC_Os04g38640	Os04g0459900 OsDegP5	B7EBF9	445	PD-PDZ	At5g39830 Cre01.g028350 Pp1s31_50V6 POPTR_0004s13440	**OsDeg8**
Os05g0147500 LOC_Os05g05480	Os05g0147500 OsDegP6	Q6ASR0	596	PD-PDZ-PDZ	At2g47940 Cre19.g752200 Pp1s8_140V6 POPTR_0014s12970 POPTR_0020s00220	**OsDeg2**
Os05g0417100 LOC_Os05g34460	Os05g0417100 OsDegP7	Q6AT72	614	PD-PDZ-PDZ	At5g36950 Cre01.g013300 Cre14.g617600 Pp1s55_7V5.1 POPTR_0008s07940	**OsDeg10**
Os05g0497700 LOC_Os05g41810	Os05g0497700 OsDegP8	Q0DH14	722^e^	NT-PD	At1g28320 Cre12.g548200 Pp1s196_28V6 POPTR_0004s04650 POPTR_0011s05510	**OsDeg15**
Os05g0568900 LOC_Os05g49380	Os05g0568900 OsDegP9	Q6AUN5	437	PD-PDZ	At3g27925 Cre02.g088400 Cre14.g630550 Cre12.g498500 Pp1s160_79V6 Pp1s198_100V6 POPTR_0001s34960	**OsDeg1**
Os06g0234100 LOC_Os06g12780	Os06g0234100 OsDegP10	Q67VA4	628	PD-PDZ-PDZ	At5g40200 Cre19.g752200 Pp1s176_87V6 Pp1s1_203V6 POPTR_0015s08440 POPTR_0004s13440	**OsDeg9.2**
Os08g0144400 LOC_Os08g04920	Os08g0144400 OsDegP11	Q7EYD8	496	NT-PD_ia_-PDZ^f^	n.a.	**OsDeg-like 2**
Os11g0246600 LOC_Os11g14170	Os11g0246600 OsDegP12	Q0ITK5	472^e^	PD-PDZ	At5g27660 Pp1s180_15V6 POPTR_0013s01900	**OsDeg14**
Os12g0141500 LOC_Os12g04740	Os12g0141500 OsDegP13	Q2QXV8	228	PD	n.a. – not a Deg?	**OsDeg-like 3**
Os12g0141600 LOC_Os12g04750	Os12g0141600 OsDegP14	Q2QXV6	593	PD_ia_-PD_ia_	n.a.	**OsDeg-like 4**
Os12g0616600 LOC_Os12g42210	Os12g0616600 OsDegP15	Q2QM57	313	PD	At4g18370 Cre02.g110600 Pp1s63_95V6 POPTR_0011s02330	**OsDeg5**
Os03g0608600 LOC_Os03g41170	Os03g0608600 expr. protein	Q75HK9	271	PD	n.a. – not a Deg?	**OsDeg-like 5**

^a^ First model identifier from the Rice Annotation Project (Build5), second identifier according to the TIGR/MSU nomenclature (Osa1 Release 6.1). ^b^ First name according to GenBank/UnitProtKB, second identifier according to the TIGR/MSU nomenclature. ^c^ If more than one protein entry was present, the different versions were analyzed by the HHPred platform (http://toolkit.tuebingen.mpg.de/hhpred/), and the one with intact protease domain and (if present) PDZ domain(s) was considered here. Sequences used in this study are supplied as Supplementary material (Additional file 1). ^d^ According to the HHPred platform. Abbreviations: aa, amino acids; n.a., not available; NT, elongated N-terminus; PD, potentially active protease domain; PD_ia_: inactive protease domain (i.e. at least one residue of the catalytic triad is mutated); PDZ, PDZ domain. ^e^ Sequence was modified based on the EST data (http://compbio.dfci.harvard.edu/tgi/plant.html) and comparison with orthologs from other species. ^f^ The HHPred platform detects secondary structures similar to RNA polymerase II large subunit from *Saccharomyces cerevisiae* in the N-terminal part of the protein – this is an indication that the predicted transcription start is incorrectly annotated.

**Table 4:** **The family of Deg/HtrA proteases in *Physcomitrella patens***

**Gene model**^**a**^	**UniProtKB acc. no.**^**b**^	**aa**	**Domain arrangement**^**c**^	**Orthologues in other plants (this study)**	**Proposed protein name**
Pp1s160_79V6	A9T3R3	500	PD-PDZ	At3g27925 Cre02.g088400 Cre14.g630550 Cre12.g498500 Os05g0568900 POPTR_0001s34960	**PpDeg1.1**
Pp1s198_100V6	A9TBD2	475	PD-PDZ	At3g27925 Cre02.g088400 Cre14.g630550 Cre12.g498500 Os05g0568900 POPTR_0001s34960	**PpDeg1.2**
Pp1s79_92V6	A9SHE2	501	PD-PDZ	At3g27925 Cre02.g088400 Cre14.g630550 Cre12.g498500 Os05g0568900 POPTR_0001s34960	**PpDeg1.3**
Pp1s21_138V6	A9RQ01	486	PD-PDZ	At3g27925 Cre02.g088400 Cre14.g630550 Cre12.g498500 Os05g0568900 POPTR_0001s34960	**PpDeg1.4**
Pp1s8_140V6	A9RGN6	618	PD-PDZ-PDZ	At2g47940 Cre19.g752200 Os05g0147500 POPTR_0014s12970 POPTR_0020s00220	**PpDeg2**
Pp1s63_95V6	A9SBN1	362	PD	At4g18370 Cre02.g110600 Os12g0616600 POPTR_0011s02330	**PpDeg5**
Pp1s237_5V6	A9TIB2	1076	PD-PDZ-PDZ- PD_ia_-PDZ-PDZ	At3g03380 Cre03.g180650 Os02g0712000 POPTR_0017s03050 POPTR_0004s08740 POPTR_0004s08720	**PpDeg7.1**
Pp1s21_327V6	A9RQ61	1072	PD-PDZ-PDZ- PD_ia_-PDZ-PDZ	At3g03380 Cre03.g180650 Os02g0712000 POPTR_0017s03050 POPTR_0004s08740 POPTR_0004s08720	**PpDeg7.2**
Pp1s31_50V6	A9RVV4	493	PD-PDZ	At5g39830 Cre01.g028350 Os04g0459900 POPTR_0004s13440	**PpDeg8**
Pp1s176_87V6	A9T734	612	PD-PDZ-PDZ	At5g40200 Cre19.g752200 Os02g0742500 Os06g0234100 POPTR_0015s08440 POPTR_0004s13440	**PpDeg9.1**
Pp1s1_203V6	A9RB23	540	PD-PDZ	At5g40200 Cre19.g752200 Os02g0742500 Os06g0234100 POPTR_0015s08440 POPTR_0004s13440	**PpDeg9.2**
Pp1s55_7V5		651	PD-PDZ-PDZ	At5g36950 Cre01.g013300 Cre14.g617600 Os05g0417100 POPTR_0008s07940	**PpDeg10**
Pp1s180_15V6	A9T7W1	473	PD-PDZ	At5g27660 Os11g0246600 POPTR_0013s01900	**PpDeg14**
Pp1s196_28V6	A9TAV2	784	NT-PD	At1g28320 Cre12.g548200 Os05g0497700 POPTR_0004s04650 POPTR_0011s05510	**PpDeg15**
Pp1s152_166V5.1		339^d^	PD-PDZ	Group 1a	**PpDeg1- group-like**
Pp1s176_111V6		527	PD_ia_-PDZ	n.a.	
Pp1s67_44V6	A9SD45	408	PD_ia_-PDZ	n.a.	

^a^ Model identifier according to Phytozome v7.0 (http://www.phytozome.net). Discrepancies between the suggested gene model and the UniprotKB entry were solved by analyzing EST data (if present) and analysis of the genomic sequence for the presence of ORFs yielding aa sequences similar to ortholog and paralog proteins, with respect to potential splicing sites. ^b^ If more than one protein entry was present, the different versions were analyzed by the HHPred platform (http://toolkit.tuebingen.mpg.de/hhpred/), and the one with intact protease domain and (if present) PDZ domain(s) was considered here. Sequences used in this study are supplied as Supplementary material (Additional file 1). ^c^ According to the HHPred platform. Abbreviations: aa, amino acids; n.a., not available; NT, elongated N-terminus; PD, potentially active protease domain; PD_(1/2)_, truncated protease domain, probably proteolytically inactive; PD_ia_, inactive protease domain (i.e. at least one residue of the catalytic triad is mutated, or protease domain is incomplete); PDZ, PDZ domain. ^d^ Fragment extended based on the EST data (asmbl_4603.p5physco4 from Phytozome 5.0., TC42496 in DCFI http://compbio.dfci.harvard.edu/cgi-bin/tgi/tc_report.pl?tc=TC42496&species=moss).

**Table 5:** **The family of Deg/HtrA proteases in *Chlamydomonas reinhardtii***

**Gene model**^**a**^	**Protein name**^**b**^	**UniProtKB acc. no.**^**c**^	**aa**	**Domain arrangement**^**d**^	**Orthologs in other plants (this study)**	**Proposed protein name**
Cre02.g088400	Deg1 Deg1A	A8I8X2	530	PD-PDZ	At3g27925 Os05g0568900 Pp1s160_80V2.1 Pp1s198_95V2.1 POPTR_0001s34960	**CrDeg1.1**
Cre14.g630550	Deg13 -	-	555	PD-PDZ	At3g27925 Os05g0568900 Pp1s160_80V2.1 Pp1s198_95V2.1 POPTR_0001s34960	**CrDeg1.2**
Cre12.g498500	Deg11 -	-	462	PD-PDZ	At3g27925 Os05g0568900 Pp1s160_80V2.1 Pp1s198_95V2.1 POPTR_0001s34960	**CrDeg1.3**
Cre02.g092000	Deg2 Deg2	A8I9B8	656	PD-PDZ-PDZ	Deg2 Group	**CrDeg2**
Cre02.g110600	Deg5 Deg5	A8I3D5	356	PD	At4g18370 Os12g0616600 Pp1s63_93V2.1 POPTR_0011s02330	**CrDeg5**
Cre03.g180650	Deg7 Deg7	A8JH35	1108	PD-PDZ-PDZ- PD_ia_-PDZ-PDZ	At3g03380 Os02g0712000 Pp1s237_5V2.1 Pp1s21_312V2.1 POPTR_0017s03050 POPTR_0004s08740 POPTR_0004s08720	**CrDeg7**
Cre01.g028350	Deg8 Deg8	A8HQB3	436	PD-PDZ	At5g39830 Os04g0459900 Pp1s31_48V2.1 POPTR_0004s13440	**CrDeg8**
Cre19.g752200^e^	- -	A8JBP6	1353	PD-betaglycan- hydrolase	At5g40200 Os02g0742500 Os06g0234100 Pp1s176_79V2.1 Pp1s1_200V2.1 POPTR_0015s08440 POPTR_0004s13440 At2g47940 Os05g0147500 Pp1s8_145V2.1 POPTR_0014s12970 POPTR_0020s00220	**CrDeg9.1**
Cre14.g617600	Deg9 Deg9	A8HNV3	619	PD-PDZ-PDZ	At5g36950 Os05g0417100 Pp1s55_7V5.1 POPTR_0008s07940	**CrDeg9.2**
Cre01.g013300	Deg10 -	-	739	PD-PDZ-PDZ	At5g36950 Os05g0417100 Pp1s55_7V5.1 POPTR_0008s07940	**CrDeg10**
Cre12.g548200	- -	A8IYE3 (fragment)	1249	NT-PD	At1g28320 Os05g0497700 Pp1s196_28V2.1 POPTR_0004s04650 POPTR_0011s05510	**CrDeg15**
Cre07.g332050	- -	A8IGX3 (fragment)	284	PD	n.a. – not a Deg?	
Cre13.g579900	- -	-	415	PD_ia_-PDZ-PDZ	n.a.	
Cre03.g203730	- CrDegO	A8IXF5	789	PD_ia_-PDZ	n.a.	
Cre38.g785300	-	A8JG98	319	PD_ia_	n.a.	

^a^ According to the Phytozome v7.0 database (http://www.phytozome.net/). ^b^ First name according the Phytozome v7.0 database, second name according to UniprotKB (http://www.uniprot.org/). ^c^ If more than one protein entry was present, the different versions were analyzed by the HHPred platform (http://toolkit.tuebingen.mpg.de/hhpred/), and the one with intact protease domain and (if present) PDZ domain(s) was considered here. Sequences used in this study are supplied as Supplementary material (Additional file 1). ^d^ According to the HHPred platform. Abbreviations: aa, amino acids; n.a., not available; NT, elongated N-terminus; PD, potantially active protease domain; PD_(1/2)_, truncated protease domain, probably proteolytically inactive; PD_ia_, inactive protease domain (i.e. at least one residue of the catalytic triad is mutated, or protease domain is incomplete); PDZ, PDZ domain. ^e^ Model is probably not correct, not supported by EST, repetetive stretches of single amino acids.

Table 1 summarizes the Deg/HtrA proteases from *A. thaliana*, which were reported before based on amino acid (aa) sequence alignments [14] (Table 1, columns 1–3). Using the HHpred platform [29], the presence of a Deg/HtrA-like protease domain could be confirmed for all of these proteins (Table 1, column 5), although two proteins seem to be proteolytically inactive. In AtDeg6 the protease domain is truncated and the protease domain of AtDeg16 lacks the Asp residue of the catalytic triad (Table 1, column 5 and Additional file 1 showing all protease sequences analyzed in this study). The remaining 14 Deg/HtrA proteases contain the conserved catalytic triad of His, Asp and Ser required for proteolytic activity (Table 1, column 5). Of the potentially active proteases, AtDeg5 and AtDeg15 (the latter with an elongated N-terminus) do not contain any recognizable PDZ domain. AtDeg7 possesses two predicted protease domains, one potentially active and a second, degenerated one with a mutated catalytic triad [6,30], as well as four PDZ domains arranged in tandems (Table 1, column 5). Considering the domain arrangement and length of AtDeg7, which is twice as long as most other Deg/HtrA family members, it was proposed that this protease arose from a gene duplication and fusion event, whereafter the second protease domain lost its proteolytic activity and acquired a new function in protein-protein interaction [30].

For the poplar tree *P. trichocarpa*, 20 *deg/htrA* genes were identified in an initial survey [16]. However, only 17 of those genes could be confirmed by this work (Table 2, columns 1–3). The discrepancy between the two studies is due to improved gene models provided by the more recent release of the *P. trichocarpa* Phytozome 7.0 database (http://www.phytozome.net). Previously described PtDeg5.2, PtDeg10.1 and PtDeg10.2 (gene models Pt792125, Pt430673 and Pt567140, respectively), [16]) are obsolete, while PtDeg14.1 and PtDeg14.2 (Pt662713 and Pt662714, respectively) are parts of a single open reading frame (ORF), designated as POPTR_0013s01900 (Table 2, columns 1–3). Additionally, a new gene model, similar to the former Pt430673 (PtDeg10.1), was identified (POPTR_0008s07940). Therefore, the genome of *P. trichocarpa* contains less *deg/htrA* protein genes than described before.

The 15 *deg/htrA* protease genes that were reported earlier for *O. sativa*[15] were confirmed in this study (Table 3, columns 1–3). However, the protease previously reported as OsDegP4 (LOC_Os03g62900) was only found in the MSU Rice Genome Annotation Project Database [27], but not in the Rice Annotation Database [26], and an additional potential OsDeg protease was identified (Os03g0608600/LOC_Os03g41170) by BLAST search and homology prediction (Table 3, columns 1–3). Both proteases lack recognizable PDZ domains. The protein Os02g0712000 (LOC_Os02g48180), originally named OsDegP2, possesses a similar domain arrangement to AtDeg7, since it contains two protease domains (a putative active and a second with mutated catalytic triad residues) and four PDZ domains (Table 3, column 5). Proteins Os01g0278600 (OsDegP1, LOC_Os01g17070), Os08g0144400 (OsDegP11, LOC_Os08g04920), and Os12g0141600 (OsDegP14, LOC_Os12g04750) appear to be proteolytically inactive due to mutated active site residues, with the latter containing two inactive protease domains and lacking a PDZ domain (Table 3, column 5, and Additional file 1).

Seventeen genes encoding for Deg/HtrA proteins are present in the genome of the moss *P. patens* (Table 4, columns 1 and 2). Two of these proteins, Pp1s176_111V6 and Pp1s67_44V6, have mutated active site residues in their protease domain and are predicted to be proteolytically inactive (see Additional file 1 for aa sequences), while Pp1s63_95V6 and Pp1s196_28V6 do not contain any detectable PDZ domain. Two other proteins, Pp1s237_5V6 and Pp1s21_327V6 have, similarly to AtDeg7, a potentially active and an inactive protease domain (Table 4, column 4).

In the genome of *C. reinhardtii* 15 *deg/htrA* genes were identified (Table 5, columns 1–3). Three of these genes, Cre38.g785300, Cre03.g203700, and Cre13.g579900.t1, encode proteolytically inactive enzymes, since at least one residue of the catalytic triad is missing in each of these proteins (column 5, see Additional file 1 for aa sequences). Cre19.g752200 contains, in addition to a Deg/HtrA protease domain, a beta-glycanhydrolase domain in the same ORF, but at present it is not clear whether this constitutes a new type of domain combination or is the result of an erroneous gene annotation. During the analysis of the Deg/HtrA sequences from *C. reinhardtii*, the occurrence of long (i.e. 10–20 aa) single aa repeats reduced the quality of sequence alignments and hints to a general problem with the assembly of the *C. reinhardtii* genome. Therefore, the number of Deg/HtrA proteases might change with future genome database updates, similar to the situation in *P. trichocarpa*.

As mentioned earlier, the number of Deg/HtrA proteases present in non-plant organisms is much lower. A general trend to an increased number of protein family members in plants has also been observed for other serine protease families [31]. However, the reasons for this phenomenon remain elusive. Compared to other organisms, plants have acquired an additional, highly structured and complex compartment, the chloroplast, and perform oxygenic photosynthesis, a process that is connected to the generation of reactive oxygen species. It is tempting to speculate that this might contribute to an increased need for proteolytic capabilities, and therefore higher protease numbers. On the other hand, although land plants are sessile and therefore cannot escape from stress conditions, the high number of genes encoding Deg/HtrA proteases is unlikely to reflect an adaptation to this life style, since the motile green algae *C. rheinhardtii* possesses a comparable number of Deg/HtrA encoding genes.

### Phylogenetic analysis of “green“Deg/HtrA proteases – proposal of a standardized nomenclature

To establish a nomenclature system based on homologies, we next examined the evolutionary relationship of the Deg/HtrA proteases retrieved from the database searches. The aa sequences of protease domains containing an intact catalytic triad as identified by the sequence alignment were phylogenetically analyzed using the maximum likelihood (ML) method. Proteases HtrA [UniProt: P73354], HhoA [UniProt: P72780], and HhoB [UniProt: P73940] from the cyanobacterium *Synechocystis* sp. PCC6803 [32] were included into the tree for comparision, due to the cyanobacterial origin of chloroplasts [33]. As the focus of this study is on green plants, no sequences from other photosynthetic eukaryotes (e.g. reg algae, diatoms) were included. Proteins lacking the catalytic triad or with an incomplete protease domain (Tables 1,2,3,4,5) were not included in this analysis to avoid misleading positions in the resulting phylogenetic tree. The presence of such inactive protease variants in plant genomes suggests that they might have acquired roles other than proteolysis, resulting in altered evolutionary pressure on the protease domain and the potential for higher mutagenesis rates.

Initial phylogentic analysis showed that four proteins, such as Os12g0141500 (LOC_Os03g62900), Os12g0141500 (LOC_Os12g04740) and Os03g0608600 (LOC_Os03g41170) from *O. sativa* and Cre07.g332050 from *C. rheinhardtii* (Tables 3 and 5) did not cluster with any other analyzed Deg/HtrA protease and seemed to be only distant relatives of this protease family (see Additional file 2 for the respective ML tree). Hence these proteases were excluded in the further analysis for clarity (see Additional file 3 for final input data).

**Figure 1:**
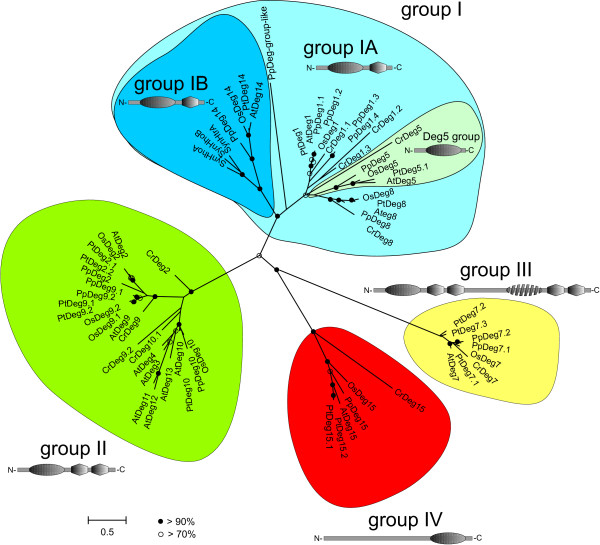
**Maximum likelihood phylogenetic tree of Deg/HtrA proteases in selected plant species.** Following plant species were investigated: *Arabidopsis thaliana, Oryza sativa, Populus trichocarpa, Physcomitrella patens, Chlamydomonas reinhardtii*, and the cyanobacterium *Synechocystis* sp. PCC6803. Phylogenetic tree labeled labeled with the new names as suggested by this study. Filled circles indicated a bootstrap support (100 replicates) of > 90%, empty circles indicate a bootstrap support of > 70%. Additionally, the domain arrangement representative for proteases from each group is indicated. Deg/HtrA proteases from clade I contain one protease domain (oval shapes) and one PDZ domain (diamonds), with the exception of Deg5 proteases, which possess a protease domain only. Proteases from clade II contain an additional PDZ domain, clade III gathers proteases with one active (oval shape) and one inactive (discontinous oval shape) protease domain and four PDZ domains, whereas enzymes from clade IV contain a single protease domain, which is shifted toward the C-terminus.

The Deg/HtrA proteases investigated here form four distinct clades (Figure 1; see Addtional file 4 for a tree containing the original gene model names), similar to an earlier study that included Deg/HtrA proteases from evolutionarily very distant taxa and only a few plant orthologs [7]. Clade I is further split into two subgroups, where subgroup IA includes orthologs of Deg1, Deg5 and Deg8 (Figure 1, Addtional file 4). Subgroup IB comprises the prokaryotic (cyanobacterial) Deg/HtrA proteases, and one protease each from the land plants *A. thaliana* (AtDeg14, Table 1), *P. trichocarpa* (PtDeg14, Table 2), *O. sativa* (OsDeg14, originally called OsDegP12, Table 3) and *P. patens* (PpDeg14, Table 4). Notably, the Deg14 protease is missing in the green alga *C. reinhardti* (Table 5).

PpDeg1-group-like (Pp1s152_166V5.1), which passed all validation procedures as described above and in the 'Methods' section, seems to be more distantly related to Deg/HtrA proteases from groups IA and IB (Figure 1). Based on its position in the tree, and the comparably low bootstrap support, it was not possible to decide whether it can be included in subgroup IA or IB. Alternatively, the gene model and the respective protein sequence might require improvement. Clade II includes AtDeg2-AtDeg4 and AtDeg9-AtDeg13 and their orthologs (Figure 1, Addtional file 4). Clades III and IV gather AtDeg15 and AtDeg7 and their orthologs, respectively (Figure 1, Addtional file 4).

Based on the phylogenetic tree, we grouped all orthologous Deg/HtrA proteases from analyzed plant species and propose a common name for enzymes from the same group in order to unify the nomenclature between different plant species (Tables 1,2,3,4,5, last two columns). Since the majority of detailed studies on plant Deg/HtrA proteases focused on *A. thaliana* enzymes, we used their well-established nomenclature [14,28] as a guideline for renaming Deg/HtrA orthologs in the other organisms analyzed here (Tables 2,3,4,5 last columns).

In *P. trichocarpa*, we renamed PtDeg5.1 (Pt771291) to PtDeg5 since only one isoform of this protein is present in this organism and combined PtDeg14.1 (Pt662713) and PtDeg14.2 (Pt662714) encoded by the same ORF (see above) under the common name PtDeg14 (Table 2). A new gene model (POPTR_0008s07940) similar to AtDeg10 was named PtDeg10.

For Deg/HtrA proteases from *O. sativa*, we propose to change the existing nomenclature present in the TIGR/MSU database [27], and we also provide preliminary new names for the more distantly related Deg/HtrA-like proteases or proteins without an intact protease domain (Table 3). For these proteins, we suggest to use the names “OsDeg-like1-6”, in order to prevent confusion between e.g. OsDeg1 (Os05g0568900, LOC_Os05g49380) and the more distantly related protein formerly know as „OsDegP1“, now OsDeg-like1 (Os01g0278600, LOC_Os01g17070) (Table 3).

Since no names were given for annotated Deg/HtrA proteases in *P. patens* we propose to name them based on phylogeny as suggested in Table 4 (last column).

For *C. reinhardtii,* the proposed nomenclature of Deg/HtrA proteases partially matched those present in the Phytozome 7.0 and UniProt databases (Table 5). However, we suggest to change the names of Deg1 (Cre02.g088400), Deg11 (Cre12.g498500) and Deg13 (Cre14.g630550) to CrDeg1.1, CrDeg1.2, and CrDeg1.3 (Table 5) since all three proteases are more closely related to AtDeg1 than to AtDeg11 or AtDeg13 (Figure 1, Addtional file 4). For Cre19.g752200, we propose the name CrDeg9.1, since its protease domain seems to be evolutionary related to AtDeg9, although the domain arrangement of this protease (it contains a beta-glycanhydrolase domain in the C-terminal half of the protein) is unusual for these enzymes (Table 5). The protease domain of Cre14.g617600, described as Deg9 in both the Phytozome 7.0 and UniProt databases, seems to be more closely related to those of Deg10 proteases, but the bootstrap support is insufficient to justify its renaming. For this reason we suggest the name CrDeg9.2 for this protein (Table 5). A new gene model Cre12.g548200 was named CrDeg15 (Table 5) since the protease domain was the closest related to those of AtDeg15 (Figure 1, Addtional file 4).

### Analysis of domain arrangement supports proposed nomenclature

Analysis of the protein aa sequences with the HHpred platform yielded predictions for the number and the arrangement of protease and PDZ domains in each Deg/HtrA protease (Figure 1 and Tables 1,2,3 and 5, column 5; Table 4, column 4). This data supports the presence of four major Deg/HtrA clades (Figure 1), as reported before [7]. Proteases from clade I contain one protease domain and one PDZ domain (with the exception of all Deg5 orthologs, where the PDZ domain is missing), whereas proteases from clade II contain one protease domain and two PDZ domains (Figure 1). Clades III and IV contain Deg/HtrA proteases with non-canonical domain arrangements: Clade III consists of very large proteins (approximately 1,000 aa), which according to prediction contain one active and one inactive protease domain, and 4 PDZ domains (Figure 1). Recently, it was shown that the inactive protease domain in AtDeg7 is involved in trimerization of this enzyme [30]. Whether this holds true for other Deg7 orthologs remains to be examined. Proteins from clade IV do not contain any detectable PDZ domain, and their protease domain is shifted towards the C-terminus (Figure 1). Since this domain arrangement is unusual for Deg/HtrA proteases [6], proteins from this group are sometimes not classified as members of this family, e.g. the mammalian ortholog of plant Deg15, called Tysnd1 [10]. However, due to the presence of a Deg/HtrA protease domain we classified Deg15 orthologs as Deg/HtrA family members (Tables 1,2,3,4,5).

Although the phylogenetic tree and, as a consequence, the standardized protease nomenclature are built on the aa sequences of the protease domains alone, they are supported by the analysis of the domain arrangements, using the aa sequence of the full-length protein. All proteases share the same domain arrangement with their nearest ortholog, e.g. all Deg1 proteins from the five analyzed organisms possess one PDZ domain, all Deg5 proteins contain none and all Deg7 proteins contain two protease and four PDZ domains (Tables 1, 2, 3 and 5, column 5; Table 4, column 4).

### A “core set“of Deg/HtrA proteases in plants

All organisms examined here contain between 15 to 17 *deg/htrA*-encoding genes, whereas the number of potentially active enzymes is slightly lower. Although the total number of Deg/HtrA proteases is similar in all plants analyzed in this study, the distribution of the proteases within the phylogenetic tree (Figure 1) differs for each species.

In the genome of *P. trichocarpa*, several genes for Deg/HtrA protease isoforms exist (e.g. PtDeg2.1 and PtDeg2.2, PtDeg7.1-7.3, PtDeg9.1 and PtDeg9.2, and PtDeg15.1 and PtDeg15.2, Figure 1 and Table 2) and this is probably the result of a whole genome duplication [34]. A similar large-scale duplication event [35] could explain the presence of duplicated Deg/HtrA protease genes in the genome of *P*. *patens* (for PpDeg2, PpDeg9, and PpDeg7, Table 4). In contrast, AtDeg3, AtDeg4, AtDeg11, AtDeg12, and AtDeg13 in *A. thaliana* seem to be duplicated versions of AtDeg10, since all of them belong to clade II and cluster exclusively with Deg10 proteases from all species investigated here (Figure 1). AtDeg3 (At1g65630) and AtDeg4 (At1g65640), as well as AtDeg11 (At3g16540) and AtDeg12 (At3g16550), are encoded by genes arranged in tandem arrays, indicating individual gene duplication events.

From this collection of Deg/HtrA protease encoding genes, we extracted the hypothetical minimum number of Deg/HtrA proteases present in plants. This “core set” represents conserved Deg/HtrA protease types found in every organism examined here, in the lowest possible copy number – for example, the genome of *P. trichocarpa* contains three *Ptdeg7* genes, however, *A. thaliana* and *O. sativa* contain only one, therefore the “core set” contains one Deg7 protease. For plants, this conserved “core set” consists of eight proteases (Table 6), such as Deg1, Deg5, and Deg8 detected in the thylakoid lumen [9]‐[17], Deg2 and Deg7 in the chloroplast stroma [18,21], Deg9 in the nucleolus [36], Deg15 in the peroxisome [8], and Deg10 is predicted to have a mitochondrial localization [14]. *C. reinhardtii*, for example, possesses only “core set” proteases as Deg/HtrA enzymes, although some are present in duplicates. This “core set” seems to provide all the proteolytic potential of Deg/HtrA proteases that is necessary for a hypothetical plant cell.

**Table 6:** Conservation of Deg/HtrA family members among photosynthetic organisms

**Organismprotease name**	**At**	**Pt**	**Os**	**Pp**	**Cr**
**Deg1**	+	+	+	1.1, 1.2, 1.3, 1.4	1.1, 1.2, 1.3
**Deg2**	+	2.1, 2.2	+	+	+
Deg3	+	-	-	-	-
Deg4	+	-	-	-	-
**Deg5**	+	+	+	+	+
Deg6	+	-	-	-	-
**Deg7**	+	7.1, 7.2, 7.3	+	7.1, 7.2	+
**Deg8**	+	+	+	+	+
**Deg9**	+	9.1, 9.2	9.1, 9.2	9.1, 9.2	9.1, 9.2
**Deg10**	+	+	+	+	+
Deg11	+	-	-	-	-
Deg12	+	-	-	-	-
Deg13	+	-	-	-	-
Deg14	+	+	+	+	-
**Deg15**	+	15.1, 15.2	+	+	+
Deg16	+	-	-	-	-
Deg17	-	17.1, 17.2, 17.3	-	-	-

The presence of a protease in a particular organism is indicated by +, its absence by -. If more than one isoform is present, the names are given. Proteases of the “core set” are depicted in bold. At, *Arabidopsis thaliana*; Cr, *Chlamydomonas reinhardtii*; Os, *Oryza sativa*; Pp, *Physcomitrella patens*; Pt, *Populus trichocarpa*.

## Conclusion

In this study, we present the first detailed analysis of the Deg/HtrA protease family in green plants, including genomes from vascular plants, a moss, and a green alga. Based on phylogenetic analysis of the protease domains and analysis of the domain arrangement in the full-length protease, we propose a standardized nomenclature for Deg/HtrA proteases in plants. Although biochemical data is only available for selected proteases from *A. thaliana*, our data suggests (within the limits of a sequence-only analysis) that proteases with the same name might indeed execute comparable physiological functions. Compared to animals and prokaryotes, the number of Deg/HtrA proteases encoded in plant genomes is much higher, which is partially due to genome or gene duplications. However, the exact reasons are probably different for every organism. A “core set” of eight protease genes was identified for plants, of which at least one copy is present in every genome examined here. This seems to be the minimum number of Deg/HtrA proteases necessary for plants. We are confident that the work presented here will be a valuable tool and guide-line for future research on plant Deg/HtrA proteases that will allow easy communication between research groups working with different photosynthetic organisms.

## Methods

### Database research

We performed BLAST searches with a peptide query against translated nucleotide collections (tBLASTn) [37] in the National Center for Biotechnology Information database (NCBI, http://blast.ncbi.nlm.nih.gov/Blast.cgi), the Phytozome 7.0 database at the DOE Joint Genome Institute (http://www.phytozome.net/), the EST-based gene indices of the TIGR database [38] (http://compbio.dfci.harvard.edu/tgi/) and with a peptide query against the protein database of Uni24rot Knowledgebase (http://www.uniprot.org/). AtDeg1-AtDeg16 (see Table 1 for accessions), *E. coli* DegP (UniProt: E0IYM0) and DegS (UniProt: E0J2L5), and human HtrA2 (UniProt: O43464) were used as query sequences.

### Analysis of sequences

The secondary structure of the aa sequences (or the translation products of the DNA sequences) retrieved by the BLAST searches was predicted using the HHpred platform, which uses a library of published crystal structures to detect domains within a given polypeptide [29]. Additionally, aa sequences were aligned with well-studied aa sequences of AtDeg1-AtDeg16 proteins using M-Coffee [39], to identify parts in the sequences derived from intron sequences in the gene model. If the presence of introns was suspected, EST-data (if present) was analyzed to improve the gene model. See Tables 1,2,3,4,5 for information about specific gene models. If the model was corrected, this improved model was again analyzed by the HHpred platform. If no Deg/HtrA protease domain was detected, and this was not due to the presence of intron sequences in the gene model, the sequence was rejected for this study.

### Alignment of protease domains and phylogenetic analysis

The aa sequences of active protease domains, as detected by the HHpred platform, were aligned using DiALIGN [40], MAFFT [41], and Muscle [42]. From these initial alignments, a consensus alignment was created by resolving discrepancies manually (Additional file 3). Gaps in this alignment were removed manually, and these sequences were subjected to phylogenetic analysis with PhyML 3.0 [43] using the ML method (default settings except 100 bootstraps in nonparametric bootstrap analysis instead of approximate likelihood ratio test). To confirm the overall topology of the obtained phylogenetic tree, the data was also analyzed by the programs Protpars (parsimony method) and Neighbor (neighbor-joining method) from the PHYLIP package [44].

## Authors’ contributions

HS and PFH designed and carried out the database search and analysis, HS drafted the manuscript. IA supervised the project and all authors edited and approved the final manuscript.

## Supplementary Material

Additional file 1 **Amino acid sequences of all proteins used in this study.** Active site residues of the catalytic triad are highlighted in red. Protease domains as identified using the HHpred platform are highlighted in cyan, PDZ domains in yellow and green. Click here for file

Additional file 2 **Maximum Likelihood tree of all Deg/HtrA proteases from this study containing intact catalytic triads.** ML phylogenetic tree of all putative Deg/HtrA proteases with intact proteases domains from *A. thaliana*, *O. sativa*, *P. trichocarpa*, *P. patens*, *C. reinhardtii*, and the cyanobacterium Synechocystis sp. PCC6803 from the original BLAST searches, using the original gene model names according to Tables 1, 2, 3, 4, 5 column 1. Filled circles indicated a bootstrap support (100 replicates) of > 90%, empty circles indicate a bootstrap support of > 70%. Click here for file

Additional file 3 **Original input data for the phylogenetic analysis.** Original aa alignment data file that was subjected to the phylogenetic analysis process. Click here for file

Additional file 4 **Maximum likelihood phylogenetic tree of Deg/HtrA proteases in selected plant species.** Following species were investigated: *Arabidopsis thaliana*, *Oryza sativa*, *Populus trichocarpa*, *Physcomitrella patens*, *Chlamydomonas reinhardtii*, and the cyanobacterium *Synechocystis* sp. PCC6803. Phylogenetic tree labeled with original gene model numbers according to Tables 1, 2, 3, 4, 5, column 1. The proteases form 4 distinct groups, labeled I-V. Filled circles indicate a bootstrap support (100 replicates) of >90%, empty circles indicate a bootstrap support of >70%. Click here for file
