# Supplementary material for: The family of Deg/HtrA proteases in plants
Source: BMC Plant Biol. 2012 Apr 20;12:52. doi: 10.1186/1471-2229-12-52 (PMC3473262; doi:10.1186/1471-2229-12-52)
Supplement: Additional file 1 — Amino acid sequences of all proteins used in this study. Active site residues of the catalytic triad are highlighted in red. Protease domains as identified using the HHpred platform are highlighted in cyan, PDZ domains in yellow and green. [file 1471-2229-12-52-S1.doc]

Additional File 1

Amino acid sequences of all proteins used in this study. Active Site residues of the catalytic triad are high-lighted in red. Protease domains as identified using the HHpred platform are high-lighted in cyan, PDZ domains in yellow and green.

>At3g27925_AtDEG1

MATTTSCSLLLSSTLFLHSPPSSHLSFFNLSSSRSSPISLYPIRSKRYFRILSKLSLNDNNRDDDDDTLHFTPFSAVKPFFLLCTSVALSFSLFAASPAVESASAFVVSTPKKLQTDELATVRLFQENTPSVVYITNLAVRQDAFTLDVLEVPQGSGSGFVWDKQGHIVTNYHVIRGASDLRVTLADQTTFDAKVVGFDQDKDVAVLRIDAPKNKLRPIPVGVSADLLVGQKVFAIGNPFGLDHTLTTGVISGLRREISSAATGRPIQDVIQTDAAINPGNSGGPLLDSSGTLIGINTAIYSPSGASSGVGFSIPVDTVGGIVDQLVRFGKVTRPILGIKFAPDQSVEQLGVSGVLVLDAPPSGPAGKAGLQSTKRDGYGRLVLGDIITSVNGTKVSNGSDLYRILDQCKVGDEVTVEVLRGDHKEKISVTLEPKPDES

>At2g47940_AtDEG2

MAASVANCCFSVLNASVKIQSSSISSPWCFVSASSLTPRASSNIKRKSSRSDSPSPILNPEKNYPGRVRDESSNPPQKMAFKAFGSPKKEKKESLSDFSRDQQTDPAKIHDASFLNAVVKVYCTHTAPDYSLPWQKQRQFTSTGSAFMIGDGKLLTNAHCVEHDTQVKVKRRGDDRKYVAKVLVRGVDCDIALLSVESEDFWKGAEPLRLGHLPRLQDSVTVVGYPLGGDTISVTKGVVSRIEVTSYAHGSSDLLGIQIDAAINPGNSGGPAFNDQGECIGVAFQVYRSEETENIGYVIPTTVVSHFLTDYERNGKYTGYPCLGVLLQKLENPALRECLKVPTNEGVLVRRVEPTSDASKVLKEGDVIVSFDDLHVGCEGTVPFRSSERIAFRYLISQKFAGDIAEIGIIRAGEHKKVQVVLRPRVHLVPYHIDGGQPSYIIVAGLVFTPLSEPLIEEECEDTIGLKLLTKARYSVARFRGEQIVILSQVLANEVNIGYEDMNNQQVLKFNGIPIRNIHHLAHLIDMCKDKYLVFEFEDNYVAVLEREASNSASLCILKDYGIPSERSADLLEPYVDPIDDTQALDQGIGDSPVSNLEIGFDGLVWA

>At1g65630_AtDEG3

MSFLCVRTVSRFRSLSRALAPGFLLLHGNAVPKTAVFFRQQSSNTRLFSSYTAPSGVEENNSKSALKNKLPPGKEVSSKDAKEKITTSAIDLALNSVVKVFTVSSKPRLFQPWQITMQSESTGSGFVISGKKILTNAHVVANQTSVKVRKHGSTTKYKAKVQAVGHECDLAILEIDNDKFWEGMNPLELGDIPSMQDTVYVVGYPKGGDTISVSKGVVSRVGPIKYSHSGTELLAIQIDAAINNGNSGGPVIMGNKVAGVAFESLCYSDSIGYIIPTPVIRHFLNAIEESGEDVSFGSINLTYQKMDNDQLRKDFKMSDKMTGILINKINPLSDVHKVLKKDDIILAIDGVPIGNDSSVHFRKKERITFKHLVSMKKPCETALLKVLREGKEYEFNSSLKSVPPLVPKRQYDKSASYYIFGGLVFLPLTKPYIDSSCVSESALGKMPKKAGEQVVIISQILEDDINTGYSIFEDFQVKKVNGVQVHNLKHLYKLVEECCTETVRMDLEKDKVITLDYKSAKKVTSKILKSLKIPSAVSEDLQPKQQNKRSKVPPKSKEH

>At1g65640_AtDEG4

MLFRFLQTLARFCRFLLISVLGFRFSPLLLLGYVKLQDENKHNSESALASGTDAKQPEAAENVTSSSIDFAVNSVVKVFTVYSMPSVLQPWRNWPQQESGGSGFVISGKKILTNAHVVADHIFLQVRKHGSPTKYKAQVRAIGHECDLAILEIDNEEFWEDMIPLELGEIPSLDESVAVFGYPTGGDSVSITKGYVSRVEYTRYAHGGTTLLAIQTDAAINPGNSGGPAIIGNKMAGVAFQKDPSADNIGYIIPTPVIKHFLTAVEENGQYGGFCTLDISYQLMENSQLRNHFKMGPEMTGILINEINPLSDAYKRLRKDDIILAIDDVLIGNDAKVTFRNKERINFNHFVSMKKLDETVLLQVLRDGKEHEFHIMVKPVPPLVPGHQYDKLPSYYIFAGFVFVPLTQPYIDSTLICNCAIKYMPEKAGEQLVLADDINAGYTDFKNLKVIKVNGVQVENLKHLTELVETCWTEDLRLDLENEKVVVLNYANAKEATSLILELHRIPSANEYDYQWQS

>At4g18370_AtDEG5

MTMALASSKAFSSIFNTLSPINQSKFVLACSGSNHVDVIDRRRRIMIFGSSLALTSSLLGSNQQRLPMESAIALEQFKEKEEELEEEEERNVNLFQKTSPSVVYIEAIELPKTSSGDILTDEENGKIEGTGSGFVWDKLGHIVTNYHVIAKLATDQFGLQRCKVSLVDAKGTRFSKEGKIVGLDPDNDLAVLKIETEGRELNPVVLGTSNDLRVGQSCFAIGNPYGYENTLTIGVVSGLGREIPSPNGKSISEAIQTDADINSGNSGGPLLDSYGHTIGVNTATFTRKGSGMSSGVNFAIPIDTVVRTVPYLIVYGTAYRDRF

>At1g51150_AtDEG6

MLFRSVHHIVARFSNSTSTPIHRFFYSPSLLRRRSSFNASLISRCCSSVSDVDVARDAVVKIFSFSREPNVVQPWQTTEKEYSSSGFAISGRRILTNAHVVGDHLYLQVRKHGSPTKYKAEVKAFRYGCDLAILGIDSEEFWEDINPLELGGIPFIGETVYALGYPRGGDTISVTKGIVTRVEPQKYSHSSIKMYVYTSGGSTNKFYSGQINKKIYDGR

>At3g03380_AtDEG7

MGDPLERLGSQASMATESVMKEDLCLEIDPPLTESVATAEDWRRALGKVVPAVVVLRTTACRAFDTESAGASYATGFIVDKRRGIILTNRHVVKPGPVVAEAMFVNREEIPIYPVYRDPVHDFGFFCYDPSAVQFLTYQEIPLAPEAASVGLEIRVVGNDSGEKVSILAGTLARLDRDAPHYKKDGYNDFNTFYMQAASGTKGGSSGSPVIDWQGRAVALNAGSKSSSASAFFLPLQRVVRALSFLQKSIDSRTDKPKAVHIPRGTLQMTFLHKGFDEIRRLGLRSETEQVVRHASPTGETGMLVVDSVVPSGPADKHLEPGDVLVRVNGTVLTQFLNLENLLDDGVGQILELEIERGGQPLSVSVSVQDLHSITPDHFLEVSGAVIHPLSYQQARNFRFPCGLAYVADPGYMLFRAGVPRHAIIKKVANEDISSLGDLVSVLSKLSRGARVPLEYMSHTDRHRKKSVLVTIDHHEWYAPPQLYTRNDSSGLWDAKPAIEPASVSPSIGNNGFPISQDISLCHHDTEPMHEVNVRGVTDIAAIMETSSGDGSQNDFGSEAKKQRVDEDSSDGIAANGSLYGSEFKSDDAMETDTTVLRDFEGATALSANASLAERAIEPALVMFEVHVPPSCSLDGVHSQHFFGTGIIIYHSSNMGLAVVDKNTVAISASDVMLSFAAFPVEIPGEVVFLHPVHNYALIAYNPSAMDPASASVIRAAELLPEPALQRGDSVYLVGLSRNLQATSRKSIVTNPCAALNIGSADSPRYRATNMEVIELDTDFGSSFSGALTDEQGRIRAIWGSFSTQVKYSSTSSEDHQFVRGIPVYAISQVLEKIITGGNGPALLINGVKRPMPLVRILEVELYPTLLSKARSFGLSDEWIQVLVKKDPVRRQVLRVKGCLAGSKAENLLEQGDMVLAVNKMPVTCFNDIEAACQTLDKGSYSDENLNLTILRQGQELELVVGTDKRDGNGTTRVINWCGCVVQDPHPAVRALGFLPEEGHGVYVTRWCHGSPAHRYGLYALQWIVEVNGKKTPDLNAFADATKELEHGQFVRIRTVHLNGKPRVLTLKQDLHYWPTWELRFDPETALWRRNILKALQ

>At5g39830_AtDEG8

MQVIASFCSKPNENEFVGRRQLLSSVCSKISQGDVVSHPPVSSVKVTQDWKSNLHELAVKSVPSTTRRILLTSLFMNLCFNPSRYLSALALGDPSVATVEDVSPTVFPAGPLFPTEGRIVQLFEKNTYSVVNIFDVTLRPQLKMTGVVEIPEGNGSGVVWDGQGYIVTNYHVIGNALSRNPSPGDVVGRVNILASDGVQKNFEGKLVGADRAKDLAVLKVDAPETLLKPIKVGQSNSLKVGQQCLAIGNPFGFDHTLTVGVISGLNRDIFSQTGVTIGGGIQTDAAINPGNSGGPLLDSKGNLIGINTAIFTQTGTSAGVGFAIPSSTVLKIVPQLIQFSKVLRAGINIELAPDPVANQLNVRNGALVLQVPGKSLAEKAGLHPTSRGFAGNIVLGDIIVAVDDKPVKNKAELMKILDEYSVGDKVTLKIKRGNEDLELKISLEEKSS

>At5g40200_AtDeg9

MKNSEKRGRKHKRQDASSAENAGGEVKEASANEASLPQSPEPVSASEANPSPSRRSRGRGKKRRLNNESEAGNQRTSSPERSRSRLHHSDTKNGDCSNGMIVSTTTESIPAAPSWETVVKVVPSMDAVVKVFCVHTEPNFSLPWQRKRQYSSGSSGFIIGGRRVLTNAHSVEHHTQVKLKKRGSDTKYLATVLAIGTECDIALLTVTDDEFWEGVSPVEFGDLPALQDAVTVVGYPIGGDTISVTSGVVSRMEILSYVHGSTELLGLQIDAAINSGNSGGPAFNDKGKCVGIAFQSLKHEDAENIGYVIPTPVIVHFIQDYEKHDKYTGFPVLGIEWQKMENPDLRKSMGMESHQKGVRIRRIEPTAPESQVLKPSDIILSFDGVNIANDGTVPFRHGERIGFSYLISQKYTGDSALVKVLRNKEILEFNIKLAIHKRLIPAHISGKPPSYFIVAGFVFTTVSVPYLRSEYGKEYEFDAPVKLLEKHLHAMAQSVDEQLVVVSQVLVSDINIGYEEIVNTQVVAFNGKPVKNLKGLAGMVENCEDEYMKFNLDYDQIVVLDTKTAKEATLDILTTHCIPSAMSDDLKTEERN

>At5g36950_AtDEG10

MLLRSFRTVELLRRISTSSVSGYRTSPSLLQRCNGFQSYLPHRVTTTESPFPSHISRFCSSQSANSQNENRHTTLSSPVSSRRVNNRKISRRRKAGKSLSISPAADAVDLALDSVVKIFTVSTSPSYFLPWQNKSQRESMGSGFVISGRKIITNAHVVADHSFVLVRKHGSSIKHRAEVQAVGHECDLAILVVDSEVFWEGMNALELGDIPFLQEAVAVVGYPQGGDNISVTKGVVSRVEPTQYVHGATQLMAIQIDAAINPGNSGGPAIMGNKVAGVAFQNLSGAENIGYIIPTPVIKHFINGVEECGKYIGFCSMGVSCQPMENGELRSGFQMSSEMTGVLVSKINPLSDAHKILKKDDVLLAFDGVPIANDGTVPFRNRERITFDHLVSMKKPDETALVKVLREGKEHEFSITLRPLQPLVPVHQFDQLPSYYIFAGFVFVPLTQPYLHEYGEDWYNTSPRTLCHRALKDLPKKAGQQLVIVSQVLMDDINTGYERLAELQVNKVNGVEVNNLRHLCQLIENCNTEKLRIDLDDESRVIVLNYQSAKIATSLILKRHRIASAISSDLLIEQNLETELASCSAV

>At3g16540_AtDEG11

MFFRPCVHTVGRYSRARVPGLLSSLFFYRSCNNVLTNSLPTVTTAGRVSRYGYICRRSSTSAAERGVFLPFALTCRRNIHSIHEDEKKLERWKKIEESHPLDELVLDSVVKVFSNSTEYSKSKPWKTLDQKSSRGTGFAIAGRKILTNAHVVMAMNDHTFVDVKRHGSQIKYKAKVQKISHECDLAILEIDSDEFWKGMNPLELGDIPPLQEVVSVVGGENICITKGLVLRVETRIYDYSDSDLLSIQIDATINDENSGGPVIMGNKVVGVVYEIGFVIPTPIIKHFITSVQESRQYSCFGSLDLSYQSLENVQIRNHFKMSHEMTGILINKINSSSGAYKILRKDDIILAIDGVPIGNDEKVPFQNKRRIDFSYLVSMKKPGEKALVKVLRNGKEYEYNISLKPVKPNFTVQQFYNVPSYYIFGGFVFVPLTKTYLDSEHHQRLADDINEGYQSLYGAQVEKVNGVEVKNLKHLCELIEECSTEDLRLEFKNHKVLVLNYESAKKATLQILERHKIKSVISKDICLPMLLDDPFKDNKINLLPWSVLPLMFDFS

>At3g16550_AtDEG12

MLFRSCVGMVSRYSRALLPTITISSRIATIVLPFALTRGRKIHTMSKDEEWWKKIRKSPPVDELMLESVVEVFTDSTKYSKVKPWQTLNQESYGGSGFAIAGKKILTNAHVVEGMNDHIFVHVKRHGSQVKYKAKVQKIAHECDLAILEIDSDEFWKGMNPLEFGDIPPLNEIVYVVGYPKAGETICVTKGVVTGVKTGNYLRSSTKLLTIHIDATTYGGNSGGPVITGDKVLGVLFQILGDKKSTGVVIPTPIIRHFITGAEESSHNAVFGSLVLSCQSMKNAQIRNHFKMSPETTGILINKINSSSGAHKILRKDDIILAIDGVPVLSEMRRISFNHFISMKKPDENILVKVLRKGKEHEYNISLKPVKPHIQVQQYYNLPSYYIFGGFVFVPLTKSYIDDKYYKITDEQHVIISQVMPDDINKGYSNFKDLQVEKVNGVKVKNLKHLRELIEGCFSKDLRLDLENDKVMVLNYESAKKATFEILERHNIKSAWASE

>At5g40560_AtDEG13

MIQLNEPLTHVRTKPILISLEFESPGRKTIDSVATESVLNSVVKINTFSSKPNICYPWQNKPQKKSKGSGFVIPGKMIITNAHVVANHILVLVIKRGSPKKYKAEVKAIGRECDLAILVIESKEFWEDMNPLELGDMPFLQESVNVIGYPTGGENISVTKGVVSRIESMDYAHGAINLPAIQTDAAMNPGNSGGPVCIGNKVVGVAFQTLGHSNNIGCLIPAPVVKHFITGVEKTGQYVGFCSLNLSYQHMDAQTRSHFKMNSEMTGILIYNINQHSDALNILKKYDVILSIDGVAIENDGTVIIPNRERTRLDDLVSLKQLGETILLKILREGKMHEFNITLRPVQRLVPAGQIDNNPSYYIFAGFVFVPLRKQHFKGSNGEQIVVISEVLADVINVEYYMYKHLKVNSVNKVKVENLKHLCELIEKCCTKDLRLELGDGRVIILDYQFAKSSTSLILERHRVPWAMSKDLMTDQSTTCSSTRLY

>At5g27660_AtDEG14

MMNFLRRAVSSSKRSELIRIISVATATSGILYASTNPDARTRVSLAIPESVRESLSLLPWQISPGLIHRPEQSLFGNFVFSSRVSPKSEAPINDEKGVSVEASDSSSKPSNGYLGRDTIANAAARIGPAVVNLSVPQGFHGISMGKSIGSGTIIDADGTILTCAHVVVDFQNIRHSSKGRVDVTLQDGRTFEGVVVNADLQSDIALVKIKSKTPLPTAKLGFSSKLRPGDWVIAVGCPLSLQNTVTAGIVSCVDRKSSDLGLGGKHREYLQTDCSINAGNSGGPLVNLDGEVIGVNIMKVLAADGLGFSVPIDSVSKIIEHFKKSGRVIRPWIGLKMVELNNLIVAQLKERDPMFPDVERGVLVPTVIPGSPADRAGFKPGDVVVRFDGKPVIEIMDDRVGKRMQVVVERSNKERVTLEVIPEEANPDM

>At1g28320_AtDEG15

MDVSKVVSFSRNFAVLVKVEGPDPKGLKMRKHAFHQYHSGNATLSASGILLPRDIFLSGEVAAKVLFEAGQDMALVLTVASVVEPFLTLGHRTSSSISQDPVKLIPGAMIEIMVEGQLKSEKESPFWVPAQLLSLVDVPVSSAALQSLIEASSGSKDSGWDIGWSLVSAANGSQPSINIEHYSKPLMQLDEPHNANFMAKSATRMAILGVPLSLLGQPSMNFASSSSKGDTLVALGSPFGILSPVNFFNSVSTGSIANSYPSGSLKKSLMIADVRCLPGMEGAPVFAKNGHLIGILIRPLRQKNSGVEIQLVVPWGAITTACSHLLLEEPSVEGKASQWGSEVLSVKSDASIPAQVAIEKAMESVCLITVNDGVWASGIILNEHGLILTNAHLLEPWRYGKGGVYGEGFKPYVLGAEEFSSTGSKFWEQKSQTLPRKAPRNHYSSVGENIREYKHNFLQTGHRDIRVRLCHLDSWTWCPANVVYICKEQLDIALLQLEYVPGKLQPITANFSSPPLGTTAHVVGHGLFGPRCGLSPSICSGVVAKVVHAKRRLNTQSISQEVAEFPAMLETTAAVHPGGSGGAVLNSSGHMIGLVTSNARHGAGTVIPHLNFSIPCAVLAPIFKFAEDMQNTTILQTLDQPSEELSSIWALMPSLSPKTEQSLPNLPKLLKDGNNKQTKGSQFAKFIAETQDMFVKPTKLSRDVIPSKL

>At5g54745_AtDeg16

MSNQVNDISYVSLIQVNIVLNKNIILWLGSATPRALRDIDLAQDSVVKIFSFSREPNVVQPWQTTEKEYSSSGFAISGRRILTNAHVVGDHSYLQVRKHGSPTKYKAEVKAFGIFGARRYTFIGETIYALGYPRDGDIISVTKGIVTRVEPQKYAHSSIEILTIQTDACINGGKSGGPVVMGNKVAGVVFENDSPSDK

> Os01g0278600_LocOs01g17070_OsDeg-like1

MTLRTGEATPPPQAHAEEEPGRGKRRRVVARETRLQQEEEEEEGASSEPETRPRPPPATPATAEEPAATAAGAEARAQLAINIRRMLAVEAERRGRIAERIGVQAEGPILTLEDTFCDIDDGSAKSQVARKVALGVSQSIVSLSSFAGRKRIRVCSGFVIRWNDSTSIGTILTSAALVRPPCGDDVRVEVFLPSGDISICQISMVDFHHNIALVEVTSNFKLQEAVILKYIIDKGDVLALGRSYEGGLLMCSRGEISNRASIFECSELLVSSCEITMAGTGGPLVNYNGHVVGINFFEENQTPFLSMAIVFKCLEHHQIFGRIIRPWIGFWFTSIQMVPLSHLEHIYRKFSDVDNGLYISNVAEGSPADIAGICQGDILMKCGGKFLSTAPEFGAMLMDKCKETMEEYDQETNGDFSAKRITVEIVIKRENDGSTIEKTISAGLIEEFNYNRWPTPIPSYKVRRDTIGRC

>Os02g0712000_LocOs02g48180_OsDeg7

MESPAKEEAGGELAMEIESTVTAEDWRRALALVVPSVVVLRTTAPRAFDTEVAGASYATGFVVDKSRGIILTNRHVVKPGPVVAEAMFVNREEIPVYPLYRDPVHDFGFFRYDPGAIKFLKYDEIPLAPEAASVGLEIRVVGNDSGEKETSLQLVGSETVDMCQSMVSILAGTLARLDREAPYYKKDGYNDFNTFYMQVRKFISYQILVWKGRYGWFVALLKFGKAASGTKGGSSGSPVVDCQGRAVALNAGSKSSSASAFFLPLERVVRALNLIRDSWEAFGSKPESDYIPRGTLQVTFQHKGFEETRRLGLRNETEQMVRLVSPSGETGMLVVDSVVPEGPAHKHLEPGDVLVRMNDEVVTQFLAMETLLDDSVGKEIDLQIERGGTPLTVKLEVEDLHSITPNHFLEVSGAVIHPLSYQQARNFRFKCGLVYVAEAGYMLSRASVPRHAIIKKLAGEDIENLGDLIACISKLSRGARVPLEYVKYTDRYRNKSVLVTIDRHEWYAPPQLYTRNDATGLWTAKSAIPPESPFIASAHHAGPIDANSNSVSSLPESSPMDLKCQHESENLTDGCIKTQTDDEINVDGSHSSEDSLVEKKRRRVDEEIAAEGTISSSGDLDEIKGGGLRHLSSVDGSDLARTISSNASLAEQVIEPALVMFEVHVPPVCMLDGVHSQHFFGTGVIIYHSDCLGLVAVDRNTVAVSISDIMLSFAAYPIEIPAEVVFLHPVHNFALVAYDPSALGAGASVVRAAKLLPEPALRRGDSVYLVGLSRSLQATSRKSIITNPCTAVNIGSADCPRYRAINMEVIELDTDFGSAFSGILTDEQGRVQALWASFSTQLKYGCSSSEDHQFVRGIPIYAISQVLEKVISGTPGPFRIINGVRRPIPFIRLLEVELYPTLLSKARSYGLSDSWVQALAKKDPVRRQVLRVKGCLAGSKAENLLEQGDMILAINKEPITCFLDIENACQKLDQSVDSDGVLNMTIFRQGKEIDLIVGTDVRDGNGTTRMVNWCGCIIQDPHSAVRALGFLPEEGHGVYVARWCHGSPVHRYGLYALQWIVEVNGKPTPDLETFIQVVKGLENGEFVRVRTVHLNGKPRVLTLKQDLHYWPTWELRGCSLGSLSMEVRKWGMRAQLPML

>Os02g0742500_LocOs02g50880_OsDeg9.1

MDDPSSTSKGKRKRGRKHKAAAENHAPASPVASTAADNPAPAAAGRRGRKSRRHEAPADADGSRPPSPPRRGEAKPVANGGGDAVVEAGGPVGWDEVARVVPSMDAVVKVFCVHTEPNFSLPWQRKRQYSSSSSGFIIGGRRVLTNAHSVEHYTQVKLKKRGSDTKYLATVLAIGTECDIALLTVDDDEFWEGVLPVEFGSLPALQDAVTVVGYPIGGDTISVTSGVVSRIEILSYVHGSTELLGLQIDAAINSGNSGGPAFNDRGKCVGIAFQSLKHEDAENIGYVIPTPVIMHFIQDYEKSGEYTGFPILGIEWQKMENPDLRKAMGMKPDQKGVRVRRVEPTAPESGCLQPSDIILSFDGIDIANDGTVPFRHGERIGFSYLVSQKYTGEKALVKVLRNSKVHEFKIKLATHKRLVAAHVKGRPPSYYIVAGFVFAAVSVPYLRSEYGKDYEYDAPVKLLVKHLHAMAQSPDEQLVVVSQVLVADINIGYEEIVNTQVLAFNGQPVKNLKNLVSMVENCKDEFLKFDLEYDQIVVLETKTAKAATQDILTTHCIPSAMSDDLKT

>LocOs03g62900_OSDeg-like6

MRLRVGTASFSLYYLSFCVFWSAGDGAHKGNDPRTKVTAKWVQGVRRMGLNLNTNPPQVDVERARQHRPSIVIVYPVKVIDGRRVVMHGAGSGFIISSTADGKCIVLTCRHVVKGSKGFDPATDLLRIRFLQGVEEDMQGQLILEDPFLDIAFILVSNMPIMLPALRFAPGVDLPVGTPVFLLGNCFLEQLPGCNIQTAIMPTIPTVSPGGISAPCKVEYGPHITRREIQFTCPNKAGYSGSPLLHEEKVIGILGRGAYQASLAVCTENLITFLETRLGVDEGTIPVEDLIQMLYQYRT

>Os04g0459900_LocOs04g38640_OsDeg8

MHCLACAAPAARAPGSRVGGGGRRRMAIECAASSPFTRDGEETAPRSMMETYGEMSSKPVLLASRRKLVALSSFCFCLHSSRYFSALALGDPSVKIDDVTPKIFPSGPLFPTEKRIAELFETNTYSVVNIFDATLRPQLNVTGVVEIPEGNGSGVVWDDSGHIVTNYHVVGNALSKKPKPGEVVARVNILAADGIQKNFEGKLVGADRSKDLAVLKVDAPTDLLKPINVGQSSALRVGQQCLAIGNPFGFDHALTVGVISGLNRDIFSQAGVTIGGGIQTDAAINPGNSGGPLLDSKGHMIGINTAIFTQTGTSAGVGFAIPSSTVLKIAPQLIQFGKVRRAGLNVEFAPDPIAYQLNVRTGSLILQVPGGSAAAKAGLVPTSRGFAGNIVLGDVIVAVDGKPIKGKSDLSRVLDDYGVGDKVSLTIQRGAETLEVTLPLEEASI

>Os05g0147500_LocOs05g05480_OsDeg2

MAGVAALFASPAFPFPSTSSVSSCSCRFRPAVARAPRHQPPGRRVTRRFDEVEGVSKRRRGIGGGGGGGSQASSSSSRKDRGLAVDFKESQVSDFEDLEEDKFLNAVVKVYCTHIAPDYGLPWQKQRQHASTGSAFMIGDGKLLTNAHCVEHDTQVKVKRRGDDKKYIAKVLARGIECDLALLSVENEEFWRGTEPLQLGRLPCLQDSVTVVGYPLGGDTISVTKGVVSRIEVTPYAHGTSDLLGIQIDAAINPGNSGGPAFNDHGECIGVAFQVFRSDEAENIGYVIPTTVVSHFLNDYQKNGKYTGFPCLGVLLQKLENPALRESLKVPSSEGVLVRRVEPTAPASKVLRKGDVITSFDGVAVGCEGTVPFRSTERIAFRYLTSQKYAGDVAQLGIIRAGNTMKVQTVLQPRKHLVPFHVEGGQPSYLIVAGLVFTPLTEPFIEEECEETLGLKLLAKARYSLSTFEGEQIVIVSQVLAHEVNIGYEHMGNQQVIKLNGTVVKNIHHLAHLVDNCKDKFLTFEFEDDFLVVLDREEATTASSDILKEHAIPSVRSSDLSEPYVDTEQEIQKPNDDFGDSPVTNYEMGVDCLLWA

>Os05g0417100_LocOs05g34460_OsDeg10

MLASVRSLRRLSSSSSSSSLSPASLRRLLLLLHRPPPPPPPPRPPLPPFQTLARGILPRIAARPVPRRFSTASCSSTLFRVGECGAPGATAIPEAERGEEEEREGEDGGEADAAVEVAAGRHDTDAYAAVELALDSVVKVFTVSSSPNYFLPWQNKAQRESMGSGFVIPGRRIITNAHVIADHTFVLVRKHGSPTKYKAEVQAVGHECDLALLKVDSEEFWDGMNSLELGDIPFLQEAVAVVGYPQGGDNISVTKGVVSRVEPTQYAHGATQLMAIQIDAAINPGNSGGPAIMGDKVAGVAFQNLSGAENIGYIIPVPVIKRFISGVEESGKYSGFCTLGISCQATENIQLRECFGMKPDLTGVLVSRINPLSDAHRVLKKDDILLEFDGVPIANDGTVPFRNRERITFDHLVSMKKPGEMAVLKVLRDGKEHELNVTVRPLQPLVPVHQFDKLPSYYIFAGFVFIPLTQPYLHEFGEDWYNNSPRRLCERALRELPKKAAEQLVILSQVLMDDINVGYERLAELQVKKVNGVEVENLKHLCSLVESCTEENLRFDLDDERVIVLKYQNAKLATSRILKRHRIPSAISADLVDEQATDDGEEATNGEIEASCTS

>Os05g0497700_LocOs05g41810_OsDeg15

MAPREVAAAARGFSAMARIVGPDPKAVKMRRHAFHLHQSGSTTLSASALLLPPGSLAEPPPLLDRICAAHGHAGGVALTSASLVEPFLVEEQRNSPSQELQPRLVPEAHLDVLVEHEESRNIGGGKTGAPRWLSARLLAIVDVQASADSVLSLLQHEGSLIRSSSWDVCWSLADVNQKQVDNDARYSLECNRKNAYAESTEPPMLAKSATRIAILGVSNLNSSNTRCINVSLMQQRGDSLLIMGSPFGILSPVHFFNSISVGVVANCLPPGTARSSLLMADVHCLPGMEGAPVFDKNSCLVGMLMKPLRQRGSSTEVQLVITWDAICNAWNSDKLERIGHPPSELVDDKSSDCKYKESCVADKHRRFVPNSANNLNQYDVSPSLTEAISSVVLVTVSETSWASGIILNKNGLIMTNAHLLEPWRFGRTSPLGLQNKIASFSEHICGGENNLLQPQQCKVSNEDAVKHELSLFNFGLKKDRAISVRLDHGERKTWCNASVVFISKGPLDVALLQMEKTPIELCAIRPEFVCPTAGSSVYVVGHGLLGPRSGLSSSLSSGVVSKIVKIPSTQHSQLSSVVEVNNMDIPVMLQTTAAVHPGASGGVLLDSLGRMVGLITSNAKHGGGSTIPHLNFSIPCKSLEMVFKYSAKGDFKILEQLDKPNEVLSSVWALAPTSSPFFSTSPENGRGGKVLEFSKFLADKQEGLKSIKDIEAFLRDRIPSKI

>Os05g0568900_LocOs05g49380_OsDeg1

MAAASSSAAACFLSPSPPPRPRHHSIKHLACAASRSPSPGPSSSRSLALPSPSASASPWPWPRRLRDLLPDETGRILSSATGSLIVALASAALILGDAGSASAFVVATPRKLQADELATVRLFQENTPSVVYITNLAVRQDAFTLDVLEVPQGSGSGFVWDKSGHIVTNFHVIRGASDLRVTLADQTVYEAQVVGFDQDKDVAVLRIKAPTDKLRPVPVGVSADLLVGQKVFAIGNPFGLDHTLTTGVISGLRREISSAATGRPIQDVIQTDAAINPGNSGGPLLDSSGNLIGVNTAIYSPSGASSGVGFSIPVDTVGGIVDQLIKFGKVTRPILGIKFAPDQSVEQLGLSGVLVLDAPPNGPAGKAGLQSTKRDSYGRLILGDIITSVNGTKVTNGSDLYRILDQCKVGEKVTVEVLRGDQKEKIPVILEPKPDES

>Os06g0234100_LocOs06g12780_OsDeg9.2

MDNHELASTSSPKRKPGRRPGRKPKPPPAPSPAAAPAPAAENGTHDPASGQKRKRGRKPKPPAAAAAAASSDGHHHPSSPLAAAVSASDSPDPASSPAPRGRGRKSRRGRPEPPSDAGAAPHAPPSPPRRGAKKGAAANAKKAAAEVPVVEPLRWEQVAKVMPSMDAVVKVFCVHTEPNFSLPWQRKRQYSSSSSGFIIGGHRVLTNAHSVEHYTQVKLKKRGSDTKYLATVLAIGTECDIAMLTVEDDEFWKGVSPLEFGSLPALQDAVTVVGYPIGGDTISVTSGVVSRIEILSYVHGSTELLGLQIDAAINSGNSGGPAFNDKGKCVGIAFQSLKHEDVENIGYVIPTPVINHFIQDYEKSGEYTGFPILGIEWQKMENPDLRKAMGMKSDQKGVRVRRVEPTAPESGCLQPSDIILSFDGIDIANDGTVPFRHGERIGFSYLISQKYTGEKAHVKILRNSKVLEFNIKLATHKRLIPAHIKGRPPSYYIVAGFVFMVVSVPYLRSEYGKDYEYDAPVKLLDKHLHAMAQSPDEQLVVVSQVLVADINIGYEEIVNIQVLSFNGKPVKNLKHLATMVEDCNEEYLKFDMDYDQLVVLEAKTAKAATQDILTTHCIPSAMSEDLRT

>Os08g0144400_LocOs08g04920_OsDeg-like2

MPPKLRSSKGVAPSPSTGEGGESHTSKKNPAPATAPTAGSVTRSRARDTTERARASGGGTALDPKKRKRTEESAASSAAAAGPVPKKMTTAGSTTLASRPLPLRYPPYPTLPPGTRPTSRQYLDPVLDRKYLDSVLEWAKERRRIAKLSKKAQCKDIPTLRHSPMDPITADAVVTSQDKAMVLRVARSVVSVSSTMPDGGGLISRCTGVVIGWDGANKRAKILTAASVVCDFHGELHNPALKLSVSMPNKTTTEGRLLFYNVHYGIALLEVMGDYKLEVPSFGSGTNYGQVIFALGRGENMSLMVSHGTISWTDYPVLLRNHNMFLSCDIPEGGSGGPVVDHGGNMIGIAFVENPGPVFISIKTIMTCMEMWDQFSRVARPLLGMQLKSVELLDVSIQEELCRDYNITSGFIVSQVLVDSTAEKLGIRRGDVIDFQDIDCSTLSQLEDHLLSLGWGYLKGMHLTVDLKVEVHNLFDSYRESITFPVQFTDASKQVEIKDEAVDHT

>Os11g0246600_LocOs11g14170_OsDeg14

MLRAARPRRAGALLLAAAAAASSSGALAYDRRGGDGDGDGDGEAFSTTTTAVRISASSPLRRALSSAASGILPGGSAHLLPSPLPLGEEDLNVYSLRAGFSFLNFFTSASNWSAGFPTQNSFASASVPPTNLSNQSSDGNSDDSKCCPGCINRNTIAKAAAAVGPAVVNISSTQETHGWVLEKSIGSGTIIDPDGTILTCAHVVLDFQSTKPILRGKVSVTLQDGREFEGTVLNADRHSDIAVVKIKSKTPLPSANLGSSSKLRPGDWVVALGCPLSLQNTVTAGIVSCVDRKSSDLGLGGIRREYLQTDCAINKGNSGGPLVNLDGEIVGVNVMKVWAADGLSFAVPIDSIVKIVENFKKNGRVVRPWLGLKMLDLNPMIIAQLKERSSSFPDVKNGVLVPMVTPGSPAEHAGFRPGDVVVEFDGKLVESIKEIIDIMGDKVGVPFKVLVKRANNVTVSLTVIPEEADSSR

>Os12g0141500_LocOs12g04740_OsDeg-like3

MDIERQVQETFSNHYKSVVLIRRGSDGIGTGFIIGKTRKSYVAMTCYHVISGNPSGALKVRLPRDTKDYVAELLYEHQGYDLAIIKVNGVSGECPILQFGDLEGVAHRANVVQLGYILGSQFALNLDPSVSPGSVIRPANQNGMMGSQDVVYSAAARHGASGSAVMFDDKVIGVLYSMSTNSQVAYARSSTTVHMALKNWLHPNDAAITTEKMIELVVKPLNDSELDD

>Os12g0141600_LocOs12g04750_OsDeg-like4

MRSGRYDLRSNLKRDEKTDFVMTYSKKHRIKEDIVCNEGEIAESLVSGDLPCESNQGVFWSELSGEVASNLSKSVVSLALHDGNTMLFVCSGIAVHRNGHVIKLLTSASLLKAFNDARKDHDNLKVEVHHEDTVVIGFLREYNLDHNMAAVIVENLPDLRPVPFNNVQKFVPHSKVVALGRDISGKLMTTSGVLIGESYNGYLMSSTCKFSEVYEGGPLFDFDGDFLGMNLSFTTEGTVFVPGDRVLDQLVNCILDHEVRFAARLEALKEVWVGESPSGEMPSSHQVHRDVLNKDRYGDLESLGYPEPPKSKDGMILAYTFEEPFGDIFGKGVWSELSEDVASSICENTVALASFNGDKRTFACTGFFIEWNECATILTSANLLRDSSDENRIAENLRIEVLLPNNLRTVGTVQHYNLHYNVALVSVKDHCVRQPVKIQPYGHNCRKLLAVGRIFESGRLMAARGQQFPTVVTHDCKFLSYSGCTTTKAGIGGPLLCFDGTFVGMNFYDEGVEGTAFLSWCEIREILKYFKTKGAVAEHSHGNPSDVLDWKIAGDDSVHPDRWPVPMPYWTLPEDLVQRKLAAKIRRQEAILC

>Os12g0616600_LocOs12g42210_OsDeg5

MAVHPLLRLLLLRPPPPPPPPPPNSPPFATTRRASSASAAAAAALLLLAASPRPRPARADPGGGGGEDIDEARVVRLFQEASPSVVFIKDLVVGRTPGRGGGQAVEAEDGEEGGATVEGTGSGFVWDTAGHIVTNYHVVAKLAGDGSAFHRCKVLLEDSSGNSYLKEGRLVGCDPSYDLAVLKVDVDGDKLRPALIGTSKGLRVGQSCFAIGNPYGYEHTLTTGVVSGLGREIPSPNGRPIRGAIQTDAAINSGNSGGPLIDSYGHVIGVNTATFTRKGTGISSGVNFAIPIDTVVQSVPNLIVYGTSVSNRF

>Os03g0608600_LOC_Os03g41170_OsDeg-like5

MDATRSAQSIHGQDEQKLYDELKSAVWVIHVESDESSGTGTGFCIDQRGLIMTCAHCVSGKTCFVARQNDKKFQKAYVLHKIESWDIAILCFVPNGSDAYPAVSLANDGTLVPGQDVYAISNQHSLMYSFCSGKVSYPCSDTVRTFDRTPRSFGKEPTDHIPSETSEYRTQKETSFTLPFNEDLPIIEMRNIHLGHGGSGGPIFLHIGKVVGMMSSGDFSKSYAVHVSALRIAFEEAKKLYSKLVNHLAASEKQSRDKNEGKNESNKSNEK

>POPTR_0001s34960_Pt706718_PtDeg1

magaasssssslistgfllchfpqfpkkkattlalsskshnnkifihnhkqhklavntttttkllrdsFLVVCTSVALSFSLLIGDVDSSALAFVITTTTPRKLQSDELATVRLFQENTPSVVYITNLAVRQDAFTLDVLEVPQGSGSGFVWDNDGHVVTNYHVIRGASDLKVTLADQSTYDAKVVGFDQDKDVAVLRVDAPKDKLRPIPVGVSADLLVGQKVFAIGNPFGLDHTLTTGVISGLRREISSAATGRPIQDVIQTDAAINPGNSGGPLLDSSGSLIGINTAIYSPSGASSGVGFSIPVDTVSGIVDQLVRFGKVTRPILGIKFAPDQSVEQLGVSGVLVLDAPTNGPAGKAGLQPTKRDAYGRLILGDIITSVNGKKVTNGSDLYRILDQCKVGEQVTVEVLRGDHKEKIPVILESKADES

>POPTR_0014s12970_Pt572750_PtDeg2.1

MAMAVANCCFSVVTSTVKFRCCVPSQPYLATSQHSVASVNCKAVVNRSRRPGEHKEGVSQKKSPGKSKDKRSSLHDEDDDGISGKRNAGKSQSMAFKSFGAQRKDKKEFKFDMKEQQFEPQNLKDAAFLDAVVKVYCTHTEPDYSLPWQKQRQYTSTGSAFMIGNGKLLTNAHCVEYYTQVKVKRRGDDTKYVAKVLARGVDCDIALLSVESEEFWEGAEPLKFGHLPRLQDAVTVVGYPLGGDTISVTKGVVSRIEVTSYAHGSSDLLGIQIDAAINPGNSGGPAFNDQGECIGVAFQVYRSEEVENIGYVIPTTVVSHFLKDFERNEKYTGFPSLGVMLQKLENPALRACLKVQSNEGVLVRRVEPTADANRVLKEGDVIVSFDDVHVGCEGTVPFRSNERIAFRYLISQKFAGDEAELGIIRAGSFMKVQVVLNPRVHLVPYHVDGGQPSYLIIAGLVFTPLSEPLIEEECEGSIGLKLLAKSRYSLARFKGEQIVILSQVLKFNGTQIKNIHHLAHLVDYNYLVVLEREAASACSSHILKDYGIPSERSSDLSEPYVDSLEDNQAVDQDFGNSTVTNLEVGFDGLLWT

>POPTR_0020s00220_Pt775566_Pt2.2

MATAVANCCFSVLTSTVKFRCCVSSQRYLATSHHSIASVTCKAVVNHKRRPSSNRDRELKEGVSQKKSSRKSKDERSYILDDDDGIRGKRKAGRSQSAAFKSFGGQRKDKNESKFDMKEQQIEPQNLKDAAFLDAVVKVYCTHTEPDYSLPWQKQRQYTSTGSAFMIGNGKLLTNAHCVEHYTQVKVKRRGDDTKYVAKVLARGVDCDIALLSVESEEFWEGAEPLEFGCLPRLQDAVTVVGYPLGGDTISVTKGVVSRIEVTSYAHGSSDLLGIQIDAAINPGNSGGPAFSDQGECIGVAFQVYRSEEVENIGYVIPTTVVSHFLNDYERTGRYTGFPSLGVLLQKLENPALRAWLKVNSNEGVLVRRVEPISDANRVLKEGDVIVSFDDVNVGCEGTVPFRSNERIAFRYLISQKFTGDVAELGIIRAGSFMKVKVVLNPRVNLVPYHVDGGQPSYLIIAGLVFTPLSEPLMEEECEDSIGLKLLAKSRYSLARFKGEQIVIVSQVLANEVNFGYEEMSNQQARFEVQWNSNKKHPSPSTPSCKNKYLVFEFEDNYLVVLEREAASASSFYILKDYGIPSERSSDLSEPYVDSLKDNQAAVQDFGNSPISNLEIGFDGLLWA

>POPTR_0011s02330_Pt771291_PtDeg5

MVVLGSIHAPSALSPLQKMTVSSSDNKSLVLGRRRTIAVGSSAVVLASLLNLHNPISNPPLLHSAFAQQQDELQQQEDRVAHLFQESSPSVVFIKDIELAKVPNRPEDRFMLTEDENAKVEGTGSGFIWDKFGHIVTNYHVVAKLATDKSGLQCCKVFLVDAGGNSLYREGTIIGFDPSYDLAVLKVDVEGYELKPATLGTSRELHVGQSCFAIGNPYGYENTLTTGVVSGLGREIPSPNGKAIRGAIQTDADINAGNSGGPLIDSYGHVIGVNTATFTRKGTGASSGVNFAIPIDTVVQYVPILIVYGTPYKDRF

>POPTR_0017s03050_Pt816849_PtDeg7.1

MGDPLERLGSETEMASLESTMKEELCMEIDPPFKESVATAEDWRKALNKVVPAVVVLRTTACRAFDTESAGASYATGFVVDKRRGIILTNRHVVKAGPVVAEAMFLNREEIPVYPIYRDPVHDFGFFRYDPGAIQFLNYEEIPLAPEAACVGLEIRVVGNDSGEKVSILAGTLARLDRDAPHYKKDGYNDFNTFYMQAASGTKGGSSGSPVIDWQGRAVALNAGSKSSSASAFFLPLERVVRALEFLQKGRNSYSNKWEAVSIPRGTLQMTFVHKGFDETRRLGLQSETEQIVRHASPLEETGMLVVDSVVPGGPAYTHLEPGDILFRVNGEVVTQFLKLENLLDDSVDQKIVLQIERGGTSLTVNLMVQDLHSITPDYFLEVSGAVIHPLSYQQARNFRFHCGLVYVSEPGYMLFRAGVPRHAIIKKFAGEEISQLDELISVLSKLSRGARVPLEYISYTDRHRRKSVLVTVDRHEWYAPPQIYTRDDSSGLWTAKPAIQPDSLQLSSAVKYMGQSVTSQTVLPSGEGTHVEHVNLGNNLELADGVTCMESSDDHSSEEPHSREESDVGTKKRRVSDLSANGIAVTDCSLSETGEVKSVDSSTMESEVSRDYQGAMTVTTNASFAESVIEPTLVMFEVHVPQSIMLDGVHSQHFFGTGVIVYHSQDLGLVAVDRNTVAISASDVMLSFAAFPIEIPGEVVFLHPVHNYALVAYDPSALGAVGASMVRAAELLPEPALRRGDSVYLVGLNRSLHATSRKSIVTNPYAALNISSADCPRYRATNMEVIELDTDFGSSFSGVLTDEQGRVQAIWGSFSTQLKFGCSTSEDHQFVRGIPVYAVSQVLDKIINGAKGPPLLINGVSRPMPLVRILEVELYPTLLSKARSFALSDHWVQALVKKDPVRRQVLRVKGCLAGSKAENLLEQGDMILAVDKEPVTCFCDIENACQALDKCSDNDGKLKLTIFRQASKWISIHMWFSGREIDLIVGTDVRDGNGTTRVINWCGCIVQDSHPAVRALGFLPEEGHGVYVARWCHGSPVHRYGLYALQWIVEINGKPTPDLDAFLNVTKELGHGEFVRVKTVHLNGKPRVLTLKQDLHYWPTWELRFDPTNAVWRRETIKGLDYSVLSE

>POPTR_0004s08740_Pt555951_PtDeg7.2

MGFQNDMPFLPEKEAFADDWAETVNKVVPAVVVLQTTTCRAFDTELPSSGSATGFVVDKQRGIILTNRHVVNPGPVNAQAIFVSNEETPLRPIYRDPVHDFGFFSYDPGAIQFLNYEEIPLAPEAASVGLEIRVIGNDSSEKVSILSGILARLDRNAPAYEKDGYNDFNTFYLQAASGTKPGSSGSPVIDKQGRAVALNAGSSSSSSSAFYLPLERVVRALRLLQKCKDARANKWETVSIPRGTLQVTFCHKGFDETRRLGLRSETEQMVRNASLLGETGMLVVDSVVPGGPAYGQLEPGDMLVSVDGELTTRFLKLETLLDDNVDQKIGLQIERGGISLTVNLTVQDLHTITPNHFLEVSGAVIHPLSYQQARNFSIQCGLVYVAEPGYMLQRSGVPCHAIIKKFSGVEISQLEELISVFSKLSRGARVPLEYIRHNDRHRAKSVIVCIDRHEWYDAPKIYTRDDSSGLWIARPAIQPKFLPLSSCSSDAEQHPKIQSFSLSGESTLAKHMHQSNKQELTNGVARIEDSNGHISKEAHSGEEYDAKTKECQEQGHFSSKEIVAANCSSREIGEIKLKDPSTTEKTVLNGIETATSTASFAESLIEPALVTLEVDVPPSCLLDGVKSVASCGTGVIVHHSQCMGLVAIDKNTVEISACDVILSFAAFPIQIPGEVVFVHPVYNFALVGYDPSALGADGASMVHAAELLPEPALCRGDRVYLVGLSKNLRAQSRKSTVTNPCLALYVHQVDRPRYGATNMEVIELDSGFGSEFTGVLCDERGKVRAIWGSFSNQDYQFVRGIPIYMISQIVDSIVCGGNGPSLLINGVKRGMPLIRTLEVSLCPMLLSEARNFGLSNDWIQALDEKDPVRRQVLCVEGSYAGSKAENVLKQSDMLLAVNKESITCFRDIENACQALEECGGSDGKLKITVFRQGCEVDLLVGTDVRDGNGTTRAISWCGCLVQDSHPAVRTLGFLPDEGYGVFVTKWSLGSPADRYCLSALKWIVQVNGKPTSDLDAFANVVKELGPDECVRVKTVDLDGKPQVQTLKQDLHYWPTWELRFDPDTAMWRKNTINALDCSNG

>POPTR_0004s08720_Pt714140_PtFeg7.3

MEDSNSREIVESEMESMDLRSQESTDQVELDSELPPEKEVITDDWRDAINKVVPAVVVLQTTACRSFDTELPSSGSATGFVVDKNRGIILTNRHVVKPGPVVAQAIFVNNEEIPVYPIYRDPVHDFGFFRYDPSAIQFHKYEEIPLAPEAASVGLEIRVIGNDSCEKVSILAGTLARLDRNAPTYRRDGYNDFNTFYMQAASGTKRGSSGSPVIDKQGRAVALNAGGSVSSSSAFYLHLERVVRALAFLQKSKDACKNKWEAVSIPRGTLQVTFLHKGFDETRRLGLPNETEQMVRQASAPGETGMLVVDSVVPCGPADRQLESGDVLVRVNGEVTTQFLKLEALLDDSVDKKIELQIERGGTSLTVDIVVQDLHLITPDCFLEVSGAVIHRLSYQQARNFCFQCGLVYVSDPGYMLSRAGIPRHAIIKKFADDEISQLEDVISVLSKLCKGDRVPLEYISYRDRHRRKSTLVTIDRHEWYDAPKIYTRDDSSGLWMARPAIQPTTLQLSPCSSNVTQGLNSQASSLNSESTPAEGTDQANNQELTHDILRTEAGYEHISEEVHSREECDVKTNKQQVQGNLSSDEIAVADHSSLEIGEMKLETPGTTEITVSNGYEGAIAAATNASFAECVVEPTLVTLEVNVPPSCLLDGIHSVHASGTGVVVHHSQDMGLVAIDKNTVETSACDVMLSFAAFPIEIPGEVVFLHPVYNYALVGYDPSALGAVGASMVRAAELLPEPALCRGDPIYLIGLSKNQRAKSRKSIVTNPYVTLNFGYADRPRYRAINMEVIELDTDFGNAFTGVLCNEHGKVQAIWGSFSNKPKSSHTTSKDHQFVRGVPIYMISQVLDKIISGANGASILINGIKRSMPLVRTLEVELCSRLLSKARSFGLGDEWIQRLVKKDPMRRQVLRVKGCLAGSNTENLLKQGDMLLAINKEPVTCFQDVENACQALENCVDSDGKLKITICRQGGEVDLLVGTDIRDGNGTTRAVNWCGCLVQDPHPAVRALGFLPGEGHGVYAAMCCRGSPADRYALGALRWIVRVNGKPTPDLDAFVNVTKGLRYDEFVRVKTINLDGKPRVLTLKQDLHYWPTWELRFDPNTARWRRETIKALDCNIE

>POPTR_0004s13440_Pt199267_PtDeg8

MLLACNRCWSLRKNCCGIIINNINDVPRHDNYSLFLGRRKLLSSSISLSSDDDVVSSYPISISSVTSGTKNLLQVICKCSPTATRRMLLASFFLFLGYHPPSRYLSAQALGDPSVTIEQVTPPVLTSGALFPVEERIVQLFEKNTYSVVNIFDVTLRPQLNVTGMVEIPEGNGSGVVWDEQGHIVTNYHVIGNALSRNPSPGQVVARVNILASEGLQKNFEGKLVGADRAKDLAVLKVEASEDLLKPIKVGQSSSLRVGQQCLAIGNPFGFDHTLTVGVISGLNRDISSQTGVTIGGGIQTDAAINPGNSGGPLLDSKGNLIGINTAIFTRTGTSAGVGFAIPSSTVLKIALQLIQFSKVVRAGLNVDIAPDLIANQLNVRNGALILQVPGNSLAAKAGLVPTTRGFAGNIVLGDVIVGVDNKTVKNKAGLDKVLDDYNVGDRVLLIIQRGSEDLEVPIILEEKS

>POPTR_0015s08440_Pt251989_PtDeg9.1

MGSDGKRKKRGRKSKTQTTLDPTTPTLNDEVFSVNNVEIVNSTTPTSANHLENRRGRPKKRPKHSPEKPPPLTNGEIGVATPVEGVARVLPAMDAVVKVFCVHTEPNFSLPWQRKRQYSSSSSGFVIRGRRVLTNAHSVEHYTQVKLKKRGSDTKYLATVLAIGTECDIALLTVNDDEFWEGVSPVEFGELPALQDAVTVVGYPIGGDTISVTSGVVSRIEILSYVHGSTELLGLQIDAAINSGNSGGPAFNDKGECVGIAFQSLKHEDAENIGYVIPTPVIKHFIQDYEKNGAYTGFPFLGIEWQKMENPDLRMAMGMKPDQKGVRIRRVDPTALESEVLQPSDIILSFDGVDIANDGTVPFRHGERIGFSYLISQKYTGDNAAIKVLRNSKTLEFVIKLSTHRRLIPPHVKGKPPSYYIIAGFVFTTVSVPYLRSEYGKEYEFEAPVKLLDKLLHSMPQSPDEQMVVVSQVLVADINIGYEDIVNTQVLAFNGKPVKNLKSLANMVENCDDEFLKFDLEYDQIVVLRMKTAKEATVDILTTHCIPSAISDDLKP

>POPTR_0012s07930_Pt823359_PtDeg9.2

MASDGKRKKRGRKSKTQTTLDPTTSTINDEVFSVNNVETVNSTTPTSAKKHENRRGRPKKRSKHSPEKPEKPPPLINGEIGVAPPVEGVTRVVPAMDAVVKVFCVHTEPNLSLPWQRKRQYSSSSSGFVIGGRRVLTNAHSVEHYTQVKLKKRGSDTKYLATVLAIGTECDIALLTVNDDEFWEGVSPVEFGELPSLQDAVTVVGYPIGGDTISVTSGVVSRIEILSYAHGSTELLGLQIDAAINSGNSGGPAFNDKGECVGIAFQSLKHEDAENIGYVIPTPVIKHFIQDYEKNGAYTGFPFLGIEWQKMENPDMRVAMGMKSDQKGVRIRRIDPTAPESEVLQPSDIILSFDGVDIANDGTVPFRHGERIGFSYLISQKYTGDNAVIKVLRNSKTLEFDIKLSTHRRLIPPHVKGKPPSYYIIAGFVFTTVSVPYLRSEYGKEYEFEAPVKLLDKLLHSMPQSPDEQIVVVSQVLVADINIGYEDIVNTQVVAFNGKPVKNLKSLANMVENFDDEFLKFDLEYDQIVVLRTKTAKEATVDILTTHGIPSAISDDLKP

>POPTR_0008s07940_Pt873612_PtDeg10

MLSSSGRAVRKLCSSSSCTAASVIRNRNLLLRERYISPLVSSNLAKYSKTTQHRAIPSLFNNFSLYSTTAEASTAAITSASAATANNHLEEASQPNLSDAYSAIELALDSVVKIFTVSSSPNYFLPWQNKSQRETMGSGFVITGKKILTNAHVVADHTFVLVRKHGSPTKYRAEVQAVGHECDLAILVVENEEFWKGMNFLELGDIPFLQEAVAVVGYPQGGDNISVTKGVVSRVEPTQYVHGASQLMAIQIDAAINPGNSGGPAIMGNKVAGVAFQNLSGAENIGYIIPVPVIKHFINGVEESGKYVGFCSMGLSCQPTENVQLRKHFGMRPEMTGVLVSKINPLSDAHRVLKTDDIILAFDGVPIANDGTVPFRNRERITFDHLVSMKKPNETASVRLLRGGEEHEFSITLRPLQPLVPVHQFDKLPSYYIFAGLVFVPLTQPYLHEYGEEWYNTSPRRLCERALKELPKKADQQLIILSQACLRFFFVLMDDINAGYERLAELQVKKVNGVEIDNLKHLCQLIRDCSSESLRFDLDDDRVIALNYQSAKVATSRILKRHRIPSAMSSDLSAEQNIPESESASSS

>POPTR_0013s01900_Pt662713+_PtDeg14

MMDYLLRKVSTCPSKYIRIPVIAIAAAAAGGSGLLYANSKHRDSDTRISLSFRAESLHESLLLPWRTPLDLTQHSWHFGNLPLFSSRISPVPSGDIKNENPGVVGESPKPSCGCLGRDTIANAAARVGPAVVNLSVPKGFYGITTGKSIGSGTIIDSNGTILTCAHVVVDFQDMRDSSKGKVDVTLQDGRTFEGTVVNADLHSDIAIVKIKSKTPLPTAKLGSSSKLRPGDWVVAMGCPLSLQNTVTAGIVSCVDRKSSDLGLGGMRREYLQTDCAINMGNSGGPLINVDGEVVGVNIMKVLAADGLSFAVPIDSIAKIMEHFKRSGRVIRPWLGLKMIDLNEMIITQLKERDPKFPNVKEGVLVPMVTPGSPADRAGFHPGDVVIKFDGKPIIEIMGDRVGKPLEVVLKRPNDVVVNLTVIPEEANPDM

>POPTR_0004s04650_Pt555773_Pt15.1

MGLPEIVDFARNFAVMVRIQGPDPKGLKMRKHAFHQYNSGKTTLSASGLLLPDTLYDADLANRILEGKSQGLGLVVTVASVIEPFLSSKHRESISQSRPELIPGAQIDVMAEGKSDLRNGADGGLDKGTSHWLRAQVIRLVDVPLSSLALQSLVEASSGSMNHGWEVGWSLASPENGSQSFMDVVQTQTEHGNASIAESQRRAREESSNPSIMGKSTTRVAILGVFLHLKDLPNFEISASSRRGDFLLAVGSPFGVLSPVHFFNSLSVGSIANCYPPRSSDISLLMADIRCLPGMEGSPVFCENSNFIGILIRPLRQKSSGAEIQLVIPWEAIALACSDLLLKEPQNAEKGIHINKENLNAVGNAYSSSSDGPFPLKHEHHISYCSSPPPVEKAMASICLITIDELVWASGVLLNDQGLILTNAHLLEPWRFGKTTVNGGEDGTKLQDPFIPPEEFPRYSEVDGHEKTQRLPPKTLNIMNSSVADESKGYKLSLSYKGPMNIRVRLDHADPWIWCDAKVVHVCKGPLDVALLQLEHVPDQLFPTKVDFECSSLGSKAYVIGHGLFGPRCGFSPSICSGAVSKVVKAKAPSYCQSVQGGYSHIPAMLETTAAVHPGGSGGAVVNSEGHMIGLVTSKARHGGGTVIPHLNFSIPCAVLAPIFDFAKDMRDISLLQNLDRPNEHLSSVWALMPPLSPKPSPPLPSLPESILQDYEKQVKGSRFAKFIAEREKLFRGTPQLGKAKSISSVIIPSKL

>POPTR_0011s05510_Pt266544_PtDeg15.2

MGLPEIVDVARNFAVLVRIQGPDPKGLKMRKHAFHQFNSGNTTLSASGLLLPDTLYDAELANRILEAKSQGLGMVVTVASVVEPFLSSKHREGISQGPPELIPGAHVDVMVEGKLGLRKDEDGVLDKGAPCWLSAQLIRLVDVPVSSLALQSLVEASSGSMDHGWEVGWSLASHESGPQPFMDTEHGNASTVESHRHARGGSSNPSIMGRLTTRVAILGVFLHLKDLPNFKILASRKRGDFLLAVGSPFGILSPVHFFNSLSVGSIANCYPPRSSDISLLMADFRCLPGMEGSPVFGENSDFIGILIRPLRQKSTGAEIQLVIPWEAIATACSDLLLKEPQNAEKGIHFNKENLNAHHNSHRPSPLPVEKAMASICLITIDEAVWASGVLLNDQGLILTNAHLLEPWRFGKTTVNGREDGTKSEDLFFPPKEFSRYSEVDGYRKSQRLPPKTMNIVDSLVADERKGYKLSLSYKGSRNIRVRLDHADPWIWCDAKVVYVCKGPLDVALLQLEHVPDQLCPTKVDFKSPSLGSKAYIIGHGLFGPRCGSSPSVCSGVVSKVVKTKAPPYCQSLQGRNSHIPAMLETTAAVHPGGSGGAVINSEGHMIGLVTSNARHGGGTVIPHLNFSIPCAVLAPIFDFAKEMRDIALLQNLDQPNEDLSSVWALMPPLPPKPTPPLSTLPESILQDNEKQVKGSRFAKFIAERDKLFRGSTQLGKAGSISNVIFPSKL

>POPTR_0018s04140_787034_PtDeg17.1

MESATSSKRQRVGNVQRAARDTEDEEEAKMFDVRSVNKDLDIDSKIAALKVSLSVVCLLSRTGGKNLCRCSGTVVECERKQESGGGGGGGEFVATILTSANLLRFPSYSRYTFLAPDITVEVYLSKGKPLKGQVLGYDFHYNLAIIQITTDYPLPTAILTDIDVSMPLTPTPLLTDSKPFGLRPHTVDSSLFKLRGGDKVIALARTLTCQCLLVESGTLSIYKSSGLHCQELLYTSCKTTQVYIGGPLINCDGEVIGIVFHYDGYAAFLPINIASRCLQLLKRDRGVCHPWLGMTLTNSYAAKASVLEEIIQKFPHISNGIIVKDVMKGSPAAHAGIVSKDIIVECDGEVVKCSLEAFS

>POPTR_0394s00220_Pt586371_PtDeg17.2

MHDYVYLSSGDTSEGEIFGFNFHYNLAIIKFKSSSRFPTAILKHIDGSIPLTTKAELQYTSFGLRPHAEPSDLFNLCPGEKVIALGRHYLSHSLMVAPGAFRPGLLRPRFSGPGFRDYDCVELLTASCRITVGGSGGPLINCNGEVIGINFYEHSYTSFLPINIASRCLECLEKNARVPQPWFGVKVTSCNAISANMFEKIIQKFGHIIEGVLVEEVIPESPACSSGVRPNDIIIRCGKQAVVSSSEFYGTLLDNTGESMEVIVMRPCLGRDLSLTIKVIDETNPEKYYRWPVPEEAF

>POPTR_0018s04150_Pt577788_PtDeg17.3

MAIRMESRDRLQGVMEKEIRSPWQRKARRRTVGCVAFRSHSFNESFDERIEENFYLDLPTKLSALKASQSVVSLVSRARRGVKNKLVIPCSGTIIGSKLEENGSFTHTILTSANLLITRAASTSEDHPPLAPEPGIKVYVYLSSGDSFEGEIFGFNFHYNIAIIKFKSSSRFPTAILKHIDGSIPLTTKAELQYTSFGLRPHAEPSDLFNLCPGEKVIALGRHYLSHSLMVAPGAFRPGVRRPGLLRPRFSGPGFRDYDCVELLTASCRITVGGSGGPLINCNGEVIGINFYEHSYTSFLPINIASRCLECLEKNARVPQPWFGVKVTSCNAISANMFEKIIQKFGHIIEGVLVEEVIPESPACSSAVLPNDIIIQCGTQGAVSSSEFYGTLLDNTGQSMEVTVMRPCLGRDLSLPIKVIDDPHPKKYYTWPVPEEAF

>Pp1s160_79V6_PpDeg1.1

MALSLATYSAHSCLSMKQQNGVIAANRTGQFSPLHGLRKQRVKNGFSGTRLVITPKLRCLKLGFAVAKAVRIVSSRGFEEVRVRGNGVKEVELRNSVEAEDGFASSSGREEGLDVESLWRAVRKRVLGGGRGWGSLVEKLITASSAAAVWMTSAAIVMGLLMSEPLDASALVLAPRKLQGDELATVQLFQENTPSVVYITNLAVRRDVFTLDVMSVPQGSGSGFIWDKKGHVVTNYHVIRGASDLRVTLGDQSVYEADVVGYDEDKDVAVLHIDAPEDKLRPLTVGSSSDLLVGQKVFAIGNPFGLDHTLTTGVISGLRREISSAATGRPIQDVIQTDAAINPGNSGGPLLDSAGNLIGINTAIYSPSGASSGVGFSIPVDTVSGIVEQIVKFGKVTRPVLGISFAPEQAVEQLGVSGVLVLDAPPNGPAGKAGLRPTTRDSYGRLVLGDVITSVNGKKIANGSDLYKILDRCKVGDMVKLEVLRGDQKVSVDVTLEPRD

>Pp1s198_100V6_PpDeg1.2

MALSLASHLDHRQLSSRKFACVSVALRCRGVPQFSSLRRTRKCSGFVSGKLEVNRKAGGGFEFGVKVPRLVRIVGGRFELGRSVEDGDGDASFSRFGESSDGVKRRWDSFEEKLAAAWSAAYLWVVPAAIVVSLMSEPLDAAAAAVLVPRKLQGDELATVELFQDNTPSVVYITNLAFRRRDVFTLDVMQVPQGSGSGFVWDKKGHIVTNYHVIRGASDLRVTMGDQTVYEADIVGYDEDKDVAVLHIDAPEEELRPLPVGTSYDLLVGQKVFAIGNPFGLDHTLTTGVISGLRREISSAATGRPIQDVIQTDAAINPGNSGGPLLDSGGNLIGINTAIYSPSGASSGVGFSIPVDTVSSIVEQIVKYGGVTRPVLGISFAPDQSVEPLGVSGVLVLDAPPNGPAGKAGLHSTRRDSYGRLVLGDVITSMNGKKISNGSDLYKILDRCKVGDTVNLEVLRGDQTVGVDVILEPRD

>Pp1s79_92V6_PpDeg1.3

MIRIRAMATLSRISLAVPLGGGGLRQNGDALRMASLVVPVSGNMVQSLARVRLFSQFRGTQLGNVKRVAPELKRGTNFEGSIRRSHGRAVAYTGEESMRETQGLESLKASQIKKVLDAGKVDYRDCLERNELVERLKDTKNFITPAAQQLLQRILCGEDDEELSASLLQEVPNLVEGWWLEAERNTVKVFQECSPSVANISTSTTATIGMSMNPVEIPRGTGSAFVWDAEGHVVTNYHVVMNGNKAKITLSDASTWEGTVVGVAKNKDLAVLKIAAPASRLRPIVVGSSQALQVGQHVLAIGNPFGLDRTLTSGIISGVGRDIRSIGGAMIRGVVQTDASINPGNSGGPLLDSQGRLIGVNTAIYSPSGASAGIGFAIPVDTVRRVVNELIRKGKVSRPGLGIMCASDSQAKQLGVNGVLVFGLSENGAAAKAGLLPTKRDLFGRIELGDIIVAINGQTLSRVDDLVAACDERQIGDRLRVTVKRGTMVRDVYITLQEIDE

> Pp1s21_138V6_PpDeg1.4

METFSVLAKAPTGISFQQIRGSLTQNGNVTKPVNVVVPKSLAKARLSSQFHGTKLRNVEFVRGESWTRRTCGVAVAFAGDGKSSQKSNDLESLKASQIKKVLDAGKVDYRDCLEKGELVKRLKDTQEFIPPSAQQLLQSYLRGEDDAELSASSVEVANPVEGWWLEAERNTVKVFQDCSPSVANITTSSTANIGLSLNPIEIPRGTGSAFVWDTDGHVVTNYHVVMNGNKAKITLADASTWEGKVIGVAKNKDLAVLKISAPAKSLKPIVVGSSQALQVGQHVLAIGNPFGLDRTLTSGIISGVGRDIRSIGGATIRGVVQTDASINPGNSGGPLLDSQGRLIGVNTAIYSPSGASAGVGFAIPVDTVRRVVNELIRKGKVSRPGLGIMCANDSQAKQLGVNGVLILGVNDNGAAAKAGLLATKRDLFGRIELGDVIVAINGNTLSRVDDLVAACDDRQIGERVRVTVKRGNMIRDVYITLQEIDE

>Pp1s8_140V6_PpDeg2

MELLVGSAATAAGAATRLTLADHHPHRISSSPCAVSLEARSLRDLTLNSSSEKPWKPKHINLQKFKRCSKISCLKHEEQQNSKRKEREKKHDRNGFEAPLSGGFNGSMEDSGFQMPDADWLYKLDDVDEGPDAPFLDAVVKvycthtdpnfslpwqkrrqysstgSGFMIQGRRLLTNAHCVEHHTQVKVKRRGDDTKFVATVLAIGPECDIALLAVDDEEFWKGVEPLKFGSLPRLQDAVTVVGYPIGGESISVTSGVVSRIEVTSYVHGASELLGVQIDAAINAGNSGGPVFHENGECVGIAFQSLKGADAENIGYVIPTTVIHHFLSDYDTNGKYTGFPSMGVLWQKLENPALRAFLKMKPDQKGVLVRRVEPTSPAFQAIKEGDVLLSFDNIPVANEGTVPFRAGERISFGFLISQKFSGDTAKCKMLRDGEVIEIETTLKAPVHLVPVHIEGKLPSYLIVAGLVFTPVCNPYLESEYGQDFEYDAPVKILEKSRHGMAEFDDEQLVVVSQVLANDVNIGYEEIANTLVKTFNGVKIRNLRHLADLIDTCTDDFMRFELDYCSLVVLETKVARSVTPKILEDNCVPTDRSQDLITSAALPLQKEEEDATANGGL

>Pp1s63_95V6_PpDeg5

MEITAASNCSRISASHCTGILESFRVEGLRIWSASSRLNSKRCSLDCRSSLRDDVVSNLKTRRRCAVVVEASLSRRSAMLSLLAASPLLFASLSHALELRSAAPKDEFDQEEESLIELFSKATRSVVSVQNVQTLGTKTVGQPVSEDDVKVEGIGSGFIWDKFGHILSHALELRSAAPKDEFDQEEESLIELFSVTSLSVVSVQNVQTLGTKTVGQPVSEDDVKVEGIGSGFIWDKFGHIVTNYHVVAKLAMDSSGWQKVQVSVLGGDGKITVHDASLIGIDSSHDLAVLKIDAPEDRLTPIPVGTSEDIRVGQNCFAIGNPYGFEHTLTTGVVSGLGREIPSPAGLPIPGAIQTDAAINAGNSGGPLLDSFGRIIGVNTATFTRAGSGMSSGVNFAISIDTVRMLVPRLIVYGT

>Pp1s237_5V6_PpDeg7.1

MKADKMNRLPTMKSESAEALKIGLTMELEPPVFDSAGSAEDWRKALNKVVPAVVVLRTTATRAFDTEVAGASYSTGFVVDKKRGILLTNRHVVKPGPIVAEAMFVNREEISVYPLYRDPVHDFGFLWFDPSLVQFLDYEEIPLAPDAAQVGLEIRVVGNDSGEKVSILAGTLARLDRDAPHYKKDGYNDFNTFYMQAASGTKGGSSGSPVIDIHGRAVALNAGSKSASASAFFLPLERVVRALHLLQAQKDKSLKGWTPAAIPRGTFQLTFSHKGFDEIRRLGLKRDTEQMVRKASPPSETGMLVVESVVPGGPAHKQLEPGDVLVRAGGEVLTQFLKYETLLDDNVGKSVILELERGGTTINVTIEVQDLHSITPNMFLEVSGGVIHALSYQQARNLHFVCGIVYVAEPGYMLSRAGVPKHAIIKKMAGEEIVNLDSFISVFFKLSKGARVPLEFVSHADRHRSKSVIVTIDRHEWYAPPRIYTRNDATGLWDIRPAAPSPEVPSVALKANHMEADGVAVHVGNGVVKEGDNGETVVIYELQVEAVTRRQKVDSELPIDLPVPTIKGKLDATSRHGSGIAPETSSSIAEHVIEPTLVMMEVHIPPSAMLDGVHAQHSFGTGVIVYHSKDLGLVAVDKNTVAISVSDVMLNFAAFPMDIPGEVVFLHPVHNYSLVAYNPRALGEVGMAAVQAAVLLPDPPLRRGDFVYLVGLSRSLQATSRKSTVTNPGEALNVAAADCPRYRALNMEVIELDTDFGHNFSGVLADEAGRVQALWGSFSKQVKYGDSSPEDHQFVRGIPIHAISEVLDVIVSGMSGGPSLVINGVKRSMPLMRILEAELYPTLLSKARSFGLSDKWVQALADKDTIRRQVLRVKGCLAGSRAHGVLDQGDMLLAISGQPVTCFRDVEIACEATKSDISGTVSPGSLDVTLFRQGREINIQVGIDDRDGFGTTRMINWAGCVLQEPHSAVRALGFLPQQGHGVYVVRWCHGSPVHRYGLYALQWIVEVNGKPTPSLQDFVDVTQELEHGAFVRVKTVYLNGKPRVLTVKQDLHYWPTWELRFDPTTAMWRRHVIKSH

>Pp1s21_327V6_PpDeg7.2

MDCSPSMKSESEDDLKLDLAMELETPVDSAGTAELWRKALKKVVPAVVVLRITTTRTFDTEVAGATHATGFVVDKQRGIILTNRHVVKPGPVVAEAMFVNREEILVYPLYRDPVHDFGFFWYDPRTIQFLDYEEIPLAPDAAAVGLEIRVVGNDSGEKVSILAGTLARLDRDAPHYKSTGYNDFNTFYMQAASGTKRGSSGSPVIDIYGQAVALNAGSRSSSASAFFLPLERVVRALHLLQEQKDMSLKGWTPAVISRGTLQLTFSHKGFHEIRRLGLKRDTEEMVRKASPSSETGMLVVDSVVPEGPAHKQLEPGDVLVSAEGEILTQFLKYETLLDDNVGKLITLKLDRGGTTINVTIEVQDLHSITPNMFLEVSGGVLHALSYQQARNLHFVCGIVYVAEPGYMLSCAGVPRHAIIKKMAGEEIDNLDKFISVFFKLSRGARVPLEFISHSDRHRIKSVLITIDRHEWYAPPRIYIRNDAFGLWDIYPAASKSEVALMALKADPMKAEADAVSAVNGTVKKGDNGEIAVKDESQVEAVKMGHKFKNEIFCDLPDPTIKGTLGSTSRQESGNAPVNSSSVAEYVIEPTLVMVEVHIPPLAMHDGVHLHHFSGTGVIVHHSKDLGLVAVDKNTVVISVSDVMLNFAAFPMEIPAEVVFLHPVHNYALVAYTPIALGEVGMAAVQAAVLSPDPPLRRGDFVYLVGLSRSLQATSRKSTVTNSTTALHVPATDIPRYRAMNMEVIELDIDLGHTFSGVLADEAGRVQALWGNFSAQAKYGNGTLQDYKIVRGIPIRAISEVLDVIVSGTSGGSSLLINGVKRSMPLIRVLEAELYPTLLSKARIFGLSDKWMQTLVERDNIRRQVLRVKGCLAGSRAFGVLEQGDMLLAINGQPVTCFRDVEISCEATKTGDSDALSPGTLDVTLFRQGQEINVKVGIDDRHGLGTTRMINWAGCILQEPHSAVRALGFFPEEGHGVYVAGYCHGSPAHRYRLHALRWIVEVNGKPTPTLQVFVDVTQELEHGAFVRVKTVNLNGKPRVLTVKQDLHYWPTWELRLDPATALWSRHNIKTHSN

>Pp1s31_50V6_PpDeg8

MPPLSQGLALNSQTLSALSLILAVPKVIASAGSEMYQDRMLSASLLQPGAVNVKSRRGFLKFCGTCTTCRRNVKKMVCKCIGEDPQCSRSNASEPLEMRNRDEIVRGGRMRRRTFGTSILGGAITLPFLQELQARALGDPTVTIDEVTPTIAPAGSLPPAEERTVELFERNTYSVVNIFDVSLRPQVNMTGSVEVPEGNGSGFIWDEEGHIVTNYHVIGSSLARNPPLGQVVARVTLLGADGYQKNFEAKLVGADKTKDLAVLDVDAPVELLRPVKLGQSSKLRVGQRCLAIGNPFGFDHTLTVGVVSGLNRDIFSQTGVVIGGGIQTDAAINPGNSGGVLLDSNGNLIGINTAIFTRTGTSAGVGFAIPIDTVAKLVPQLIAYGKVMRPGLNVQFAPEAVAKQLNVRSGALVLTVPDKSAAAKAGIIATRRGLTGNILLGDVIVGVGETTVKNPQELTKALDSYQVGDQILLKVQRNDQIQVLPLTLEESMR

>Pp1s176_87V6_PpDeg9.1

MEPPPRKRGRPLKHPKAAPSDSLKSRVASDLNEYNSLSPSTPVPVDLADPAVSPVDPRSGQRGRKKPVAEKVEGLAPITPDGGGGSSSDRELSKSVGDDSELPAEGVARKRNKKSSLGNADDIKGHAPSLEDATGGRDDDLKDAPFMDAVVKVFCVHTEPNFSLPWQRKRQFSSNSSGFIIKGRRVLTNAHSVEHHTQVKVKKRGSDTKYLATVLAIGTECDIAMLTVSNDEFWEDVTPVNFGSLPRLQDGVTVVGYPIGGDTISVTSGVVSRIEVTSYVHGATELLGVQIDAAINAGNSGGPAFNDRGECVGIAFQSLKHEDAENIGYVIPTPVIDHFITDYVRNNDYTGFPILGIEWQKMENPDLRKALGMKSTQKGVRIRRVEPTAPAFAQLRASDILMSFDGIDIANDGTVPFRHGERIGFSYLVSKKYSGECAKVKILRDGKSKEFDIDLVNHKRLVPAHIKGKPPSYYILAGIVFAAISVPYLRSEYGKDYDYDAPVKLLDKLLHSMSQSEDEQLVVVSQVLVADINIGYEDIVNTQVVAFNDTPVRNLKHLANLVEKCTDPFLRFGLDYQQIVILETQTAKAATPEILATHCIPSAMSHDLKVEC

>Pp1s1_203V6_PpDeg9.2

MEAPPRKRGRPPKHPKVASNDSPKSRANSAFNSVKSPSASSPADVEMVDRGVATVDSKSVQRGRKKSAGDKVEGAASVKPSGGGGSPSDRELSKSEADDVELLPEKLSSRRNKKQSLANPGLFSNDNTRGHAPLFEDGTVGSEDVKDAPFMDAVVKVFCVHTEPNFSLPWQRKRQFSSNSSGFIIKDRRVLTNAHSVEHHTQVKVKKRGSDTKYLATVLAIGTECDIAMLTVSNDEFWEDVTPVDFGSLPRLQDAVTVVGYPIGGDTISVTSGVVSRIEVTSYVHGATELLGVQIDAAINAGNSGGPAFNDRGECVGIAFQSLKHEDAENIGYVIPTPVITHFITDYVRSGDYTGFPILGIEWQKMENPDLRKALGMKGIQKGVRIRRVEPTAPAYKYLQASDILLSFDAEDIANDGTGVWQYGKDYDYDAPVKLLDKLLHSMSQCDDEQLVVVSQVLVADINIGYEDIVNTQVIAFNGTPVRNLKHLAIMVEKCTDPFLRFDLEYQQIVVLETNAAKAATPEILATHCIPSAMSDDLKT

>Pp1s55_7V6_PpDeg10

MKSVPVWRTLYAIRGLTTSNRNAGKTRTLNADGRRIQRLSENCSGVARESRITQSLISQGVLVGSGNLDDFRAGDRTSNGQCPVDHMEFSSFFRGPWSNSIRARGFSTNIANSAVERSRTQDPYLATEIALDAVVKIFAVSSSPNYFLPWQNKAQREVTGSGFVISGRRILTNAHVVADQTHMMVRKHGSPIKYHAHIEAVGHECDLALVRVHDDEFWEGMLELELGDIPFLQESVAVVGYPQGGDNISVTGGVVSRVEPTRYVHGAAHLMAIQIDAAINPGNSGGPALMEDKVVGVAFQNLANAENIGYIIPVPVIKHFLADVEETGDYIGFCSLGITCQSTENVQLREHLKMPAGLTGVLVNKIHPLTDTSRCLKRDDVILAFDGTPIANDGTVSMKKAGEAAKVTVLRAGEQLHFDIKLGPLRPLVPIHQFDVLPSYYIFAGLVFTPLTQPYLQEYGEDWYNTSPRRLCERALSAYPTEPGQQLVILSQILMDEINTGYERFHDFQGMCFKVNKVNGIEVHNLRHLRKLVEECTETSIRFDLDVNRVLVLDFRAAREASLRILQRHRIPSHTSKDLLEEEDSKDSAFLSWEGPWHKEENKDSEGIPIEELEVPEPVKVDDTSLGKRLVNRQISTPILNFSKLHQRIYL

>Pp1s180_15V6_PpDeg14

MAVRWSSRTRQSLKFIGKLSNEAPSKPLRSSGELIHSFECKANPSDRFGLRSAVVASLVFCTTSGALYTEQLNGRPERDGIGGGVTTPSHEGESGRFNEPRRTLDLPHSGDNGCNLNNRTPIVRVSNASPFPGSAAKSPSTKGASLPSKQGGCLSRYAIADAAAKAAPAVVNVKVSLGERNAFFGETAGSGFIIQSDGTILTNAHVVATDRRGLHKGSLIVTLQDGRNFEGEVVSFDSLSDLAVIKVNSSRPLPVVKLGSSKDLRPGEWVVALGSPLHLQNTITAGIISCVDRKSSEIGLEGVGTGYIQTDAAINQGNSGGPLLNLDGEVIGINTMKALAADGVSFAIPIDSAIKIVDQLKKRRHVVRPWLGMKMWELTEPRISQLKERRPGFPDVNAGILVSQVIPGSPAFRAGVLNDDVIIEFNGVPVTTIDQIVEALGDKVGTSFKMVVKRRNNEQVVLHVTAEEASPDL

> Pp1s196_28V_PpDeg15

MNMKKPGSTALGQADLYETTSSEPSVVVITCASIVEPFLAPKSHGISSREDFPKLIRGAEVDILVEVPDLSEKGSGYSGEHSVCWLPGQLLAVVDVPAAGAALQDLLDVHGGSVKGVWEVGWALAPVEDNAQQLHSLLTSEILTETQLQEDVASQHDLKGDKRPVAHAVAGVSRLGMAAAAATKIAVLSVGSHGDSSALNTCLKKRHQLSKVSNGLSKMPTGHVIAEQKRRGDSLIAVGSPFGALSPLHFQNSVSVGIVSNLWPPTRGPPSLLMADVRCLPGMEGGPVFDERGNLVGMLTRPLRQRGGAAEVQDVTHFVANMHMQLVMTTDVLLPVLQRVGINVGVLCNTKSPDVQLQPSASAMLVENSSFETQPCRPGSIESHSTYQAMQYVPSAVERAVTSVVLITIGDGAWASGVILNKTGLILTNAHLLEPWRFGKPRMVPSPVNGSIPKDSGFPLSCDESQEDLALNQLQEEEISKGVSTSRTSSWPTDVSQKNYQRIRVRLDHRQPRSWHAARPVYVSQGPLDIALLQLESPPPGLHPITPDKECPTPGSTAVVLGHGLFGPRSELRPSVSAGVVARVVKAGSSPFLTGIGGRKEGKASKAAMLQTTAAVHPGGSGGAVVSGEGHMIGLVTSNARHSGGTVIPFLNFSVPYAALVPVFEFASSADSDWSRLEELDKPNDQLAAVWALVPPTPPRPSPPPFFRRHPRVPVSQEIDPDKGVPKGSRFAEFIKEKGGLDWKSPKSLPPPLPEESPHRGSPPCHYEEPSYAARASFPIHSRL

>Pp1s152_167V6

MFVDVNKSSGKKVRIINRPGAQLIIGHRPARPHVSASPIVISPAPAIQYNMEDITRKCKESVVHIMASTEKMSSSGGSVWDTHGHIITNNHVLISDNLKVTLPDGTKYEAVVVKRYKSQDLAMIKLRGCCSKLVPVVTCSEISPYALNVGNFVFAIGFPLSCEMTVSYGHVSCFPEQPINVDGSLVRDAIQLDANINHGNSGGPIFNSRGECVGVIVCGFRNSGINYGIPLKTVTNMVKRFKEESAEQKGEAILPNLKPFLRDIKNTMQELSIRTGYQTGPASGGLHSLASSSTTRSIVHCTKLKYRSLPCSCSMCRHLVPSEQPGLVKGYSKRVYPPA

>Pp1s176_111V6

MLTVSNDEFWEDVTPVNFGSLPRLQDGVTVVGYPIGGDTILLTSGVVSRIEVTSYVHGATELLGVQVRSAVLQACAQFGNYVVSSMNVRHVHLSMRVWPGPNNSGVTLPEGPDVARGQCTWLVGGSGRSEDWEIDCAGSNEGRNDVEMTLVPFCVDLGKYMFSFTLHLFSQRTAVARVSLVLGLWDLFWGACGELLDLLAFLVGMQIDAAINGGNSGGPAFNDRGECVGIAFQSLKHEDAENIGYVVPTPVIDHFITDYVRNNDYTGFPILGIEWQKMENPDLRKALGMKSTKNGVRIRRVEPTAPAFAQLRASDILMSFDGIDIANDGTVPFRHGERIGFSYLVSKKYSGECAKGKPPSYYILAGIVFAAISVPYLRSEVRVQWNMIWWDVEDEQGVMVKVARDSLTVYGKDYDYDAPVKLLDKLLHSMSQSEDEQLVVVSQVLVADINIGYEDIVNTQVVWFNGIPVRNLKHLATLVEKCTDPFLWFDLDYQQVLVLETQAAKAATPEILATHCIPSAMSHDLKI

>Pp1s67_44V6

MRVSGTTLGQAEAVAARDPATPGMESTLTGGGRPSSEPRMKPTANAERFLEGEHNKAVLQLDGTMLHREESEVTTTTPATEEAAMREKPTQKEKANYKITEICVLHAYRYSWLYQEALLGAEESIKETRKFMTFGRNFSKKLMTSPYIRINENLDEGVMSTLPRPTSKSTEIFTNIGYLTVERGRAQDPHLAADIALSAHVKIFAVSRSSKVFLPWKNQSSHRTIGSGNSGGSALKENKVVGVVRKNTSKGRKQKILVRTLQSVALDLHFGCHDRDDVILFMDDVPIAYDGRGNGFVRSQGVRSEPVTIRYHLTPTSNDFVQLQHLVPIHQFGMLLRHYILADLAFIPLTEPYLQEYVKDFYNTSPRRLSKRLPSDFPTKTSQQFFIFAQMEIEGWHGEFLGQKLICE

>Cre02.g088400_CrDeg1.1

MHNLHSRCSTAPSRPKNASGWGTYAGSARQRLQRSRVHVPSWVWLGQGQSTANQSSGAAASCDEVVTTVSAAAPMTNGDQGCSRTFRHVPVTPGRNPVMEPEASQSPRERFLSGAVALLGTASLLVGAVTVGPSPVMAKPRMTPEEQLTIEIFRKNTPSVVNVTNLAVKRDAFTMNMLELPQGQGSGFIWDATGHVVTNYHVIQDASDIKVTLSGGEEFSAKVVGVDQDKDIAVLQIGPIGPPASEAQGDGSSKPPQQLLAAAPGAAAPPSAAVPPPSASSLPPGAVPVAPPPPEPVRVTPLSLCSSSADIVVGQKVFAIGNPFGLDHTLTTGVVSGTGREIQSISGRPIQDVIQTDAAINPGNSGGPLLDSGGCLIGINTAIYSPTGANNGVGFAIPVDIVKSSVGQIIQYGKVTRPILGISFAPDQSSEALGIKAGILVLSAREGGPAWRAGLKGSSRDEYGRLVLGDIITAVNGVKIKTSSDLYRVLDKSAVGDTLRIQVLRENTTFEVNVVLDSNNPPPQPEQPKA

>Cre14.g630550_CrDeg1.2

MKALRSLRGPSAAANGSSSSAGVASGSSSGGGAASGSSSGGGAAAASPVLGELSLSYEPTSGLAFSGAGAASATAFASSSAGAGALTRSGYSQGYSQGGSGSSSTSGGGGGGSSGTFTYGAASGYGSQQQQQQGLQPASVAAGVGSSSNRGNSSSLSSGLAASGVSTSADAGAAAPAAAGGGAAATDGAAAGGGTSSSSGGGGGSASGGGSAAALPPGVAAEDVALASALGLGVGEAAVVRLFERWRPSVVNISGMRAMQASGVVWGDRGCVVTAYHLVKGAAEVKVTLYDNSSYTAKVLGHDAAKDIAVLKLSVPKSKLRELQPVSPGSAAGLRVGQCVYGIANPWGLGHTLSQGLVSGLGCELSGCGLLPLKGVIAATTGLGPGSDPGSSGGVLLDSRGAVVGMLVSPPASGGGGGGGGGRSYAVPLDAVRGLVAQILSYGRTVRPALGITMAPPQVLERVGVEGVLVLEVPPGSPAHAAGLRPTHRDIFGDLVLGDVVVGLDGKPVRSAADLYDILDEHRVGDRIKLDVLRDGKATGLTVTLGERVLGGSEE

>Cre12.g498500_CrDeg1.3

MAVSASATQRSGPAGSEPADQPRIGPLALAAGTALCLMLGSGFAPEGSSGALAPLGALAVRPAHAEATTLKGTPMASRAADLTAPSVRDAVPLQRNIDPLTMQLPDAPPDLTPEEVRAIRIFARNTPSVVNITNFRQVPVQSVYGFGRGGFSMDTEKVPAGLGSGFVWDDKGHIVTNYHVIKGADEVKVTLLDQTTYTAKVVGGDADKDVAVLRLVDAGDKISALTPVTLGSSAQLLVGQRVFAIGNPLGLEHTLTSGIVSGLNRELSTGTLTIKGLVQTDAAINPGNSGGVLLDSAGRVIGINTAIADPTGKGASSGVGFALPIDSVRGLVDQILTYGRVLRPVLGVSLAPPQVLKQLGQPGVLVLEVPKGSPAEKAGIKPTMRDRFSGSLVLGDIITGIDGKAVKNYSDLVEALDEKRVGDTVKVDILRSQTDNLFASGTARKMSVYITLGERGQVNVTE

>Cre02.g092000_CrDeg2

MAAPAPQSSRSGFGSAAGLQPHLTNSVCTVMPPPSPSKRMRSDSFDHIDPRALPAPAAAAKPAAAAATDPEAALSKALKSVVKIFCTSANPNYALPWQMMAQSKSTATGFVVAPLNSRRILTNAHAVTNQVQVMLRKHGNARKYPARVLAVGHECDIAMLTVDNDEFWTGDMEALEVGQLPSMQEAVMVVGFPTGGDNVCVTKGVVSRLDRQVYSHGRCALLTTQTDSAINSGNSGGPVLQGAGKLAGIAFQSLIGAENTGYVIPVPVVNHFLTDLERHGGRYTGFPEMGMSWQTLESTSMKDSLKLPRGATGVYITSTDPCYNASKELRVGDVLTHVQGHSIADDGTFLFEGQNVRIDFRHLSSMAYDGESLQLRVWRDGAAHELSVQVSVPKHLVLPHCHDLKPRYFIYAGLVFTRLTNFYLRHQYGADWSTKAPIKLCDRYYGGVMEAPGQEVVLLSKVLSADVNQGFQDLQNYQVYKVNGVKVHNLQHLAQLVERCESDYIRFELDWKRASLYIKCPCVRDTVVVLHTASGRAATSEILKMNAIASACSEGLMEEPLCLPPDLEATAQPSLADSEVVPDVAAEPIDADGGASGKDGSNGNNGSNNGMTAGGAASSSKGVQPPAGSSVPSSGAGAGMDTASAAVAMANGWARL

>Cre02.g110600_CrDeg5

MPAQDLAAPHLKYYRSNAWQGRCWHASSLRSGAQTCIRAWGPHTHSIAPQQPCPSPPHASSVASTSSSSISRRGVLLAQLAAAGLVGMPSRQAQAASFVDDAISTAVYNASAPSVVSIAIVRAKSGIPTREPIGSGIVWDTAGPHVVTNFHIIPPLNSTNSLLEVTVTDLASGTVHTLGARVAGTDSIHDLAVLQLLPRAGDTTAGAAEGEPGASGAAAAWVPALAPIRLGTSADLRVGQTVYALGCLPSSSSSPQTPALTTTMSVGLVSGLQRSIPSPVGARIYGVLQTDAVINAANSGGPLLDSAGRLVGLNAAVGRSQTVSARGSGVGFALPADLLFDVVPKIILYGNPYGKK

>Cre03.g180650_CrDeg7

MSLEETEPLVEPTTRGSPEDWKRSLEKVVPCCVVLKVTQTRAFDTEAAGSAYATGFIVDKQRGLILTNRHVVTPGPIVAEAIFLNREELPVVPLYYDPIHDFGFLRFDPSRLQFMEVSEVPLAPEGATVGLDIRVVGNDSGEKVSILAGTLARLDRDAPVYGRKGYNDFNTFYLQAASGTKGGSSGSPVIDCQGRAVGLNAGGKNKAASAYYLPLERVVRALKLIQASKDACDKPGVWPAPVIPRGDLQTTFVFKGFDEVRRLGLQQETERRVRNSKTAPGPEGAQHSTGMLVVDSVVPGCACDGVIEAGDVLVELNGQVVTHFLALEELLDDAAQDRMGGGSGKVQLLLERGGKPIRAEVDVTDLHTVTPNCFLELAGGAVHALSYQQARNNRAVVGQVYVAEPGYMLGRANVPKCAIITGLNGKPTPDLITFATVLRTLPHGARAPLEYLTFGERHRRKNAILHVDRQWYGPPVYWTRDYALGTWHPTTDYPPGAPPPPEPSIKPLAASTAEPISTEARLANGHATGTADASPAPMDTEQVAAANGGGGAAAASTSGREGSTPAAGTDSGAGDMQSDDLDELLRCCLVLVDVDIPLVALSDGVHSRSFAGNGLVVYAGERVGLVLVDRNTVAVGPCDVNLSFGAHPAEISGRVRFLHPLHNFALVSYDPAALPPEARAKVRAAELLPYPPLKRGEAMRLVGLTKHLRVMQRTSTVTNATAALTIPSAEVPRFRAVHEEVIKLDQDFGATFSGCLTDNRGRVRGLWCSYSEQVEKEEREWCAGLHAAVFYPWVEQLARQLDQDAPAPPPTACVLDAELEAVLLSKAAQFGLPGEWVSRLDQLDPERRQVLRVRSCVAQSHASKVLRSGDMLLAMSGRPVTCFQDVERLISEASAAAATAGAAGTGPAAEPTEAGTVDEPASKRPRVDAASEGAAAAAAPADGSGRPTMHLTIYRSGAVQDVEVVLGQEDGMGTGRLVHWCGAQLQAPHRGVRELGFLPEGAAGVYISRWHHGSPAHRYGLYALHWIQQVNGVDTPDLDSFLAAVANIGDGQFVRLKVCHLETTQPKVLTLKLDLHYWPTWELRLDPGTCSWRRLQHGKLGTAKQAAA

>Cre01.g028350_CrDeg8

MERAFKTRTSGQGARPCQRCSRFTRSAVCVQALGRGEDHELNCTTRRGFVARVLGGAVAGGVGAVLGDTRPAGALTLEQVTPTVVPAPALPPREAAIVSAFERANYSIVNVVDLLLPGRAAANPEVDIPEGNGTGLIWDGEGHVVTNYHVLLNSLKGLSGPNPAANRPKVAKVTLLNAAEGGLEQTLDAVLVGVDRTRDLAVLQLVAPPAGALRPAALGSSATLRVGQQCLAIGNPFGFSHTLTTGVISALNRDIKSQLGTTIPGGIQTDAAINPGNSGGPLLDSSGAVIGINTAIFTPSGSSAGVGFAIPVDMVKSVVPQLIANGRVVRPSLDAQIAADTVAARLNVGRGALIQAVTAGGAAEKAGLLPTRRGLSGIVAGDVIQAINGRAVNSAGDLLVALDGLAAGERAELRIVRSTDQGLQELSVAVTLSAEK

>Cre19.g752200_CrDeg9.1

MTSLLSALGRRASAGTPLGRPAPGGSTLPAVAPAAAASPALPRRTCCPTAALSTTASSACGAAPGISAEPLPQLASCPSRISSSRSGGRRSRVLLAAPPRSTGNGQVGPPQHRKGVVAPGAGAGAAAASAAAPAAAVAAAVDEEVADDDEDVLMDEDEAGQQLAPFMDAVVKVYCMHTEPNYSLPWQRKRQYSSSSSGFVVSHGGRNWLLTNAHSVKVKRRGDDRKFLARVISVGVDCDIAALQVDDPDFWAPQSPDSPPPPVLELGPLPRLQDGVAVVGYPIGGDTISVTAGVVSRIEVTDYSHGSTDLLAIQIDAAINGGNSGGPVFNRACQCVGIAFQALVGSDVENVGYVIPTPVVAHFLDDYTRTGGFSGFPQLGIQWQRMESEALRRADLRESYLPAFRACVVEGRAHSVMCAYNKVNGVPACAHPHLLRATLNDSWGFGSDPANFVVSDCGAVSDLALTHGWNTSLAAAAAEALTAGGLSLFCDNAAAAAVPQAVASGLLAPAVLRAAVRRMLLARCRLGILGAAQPNTTNTTDSSSSTQLDTSEAPAGASSTSADGTLSQAEAAVDRSGNSGSGGGSGSGSGGGSGSSSGSGSGSGGGSGSSSGSGSGSGSGRPDVQALSRSHAHTRLAYEAALRSITLLVNRPPPGGSGGRPLLPLQLPPPPPAPANTTDNTTAAPGPLLALLGPHADGALYYLGTYYGTPSHPVVTPLAALREALGPAAVSHTPCLTGVGLEPSDGLHTCTQAAAAAQVAAVVFVGGSSRNFACGDQQCTTITPVSESEGLDRGSLRLPGLQEELVRAVARSGVPVVVVAVAGGPLDLSPLLGLQGVAAVLAAPYGGQQAGYALASVLLGTSSPSGRLPATWLYDWYTHLSDPTSMAMRAWPGRTYRYLQGGYPLASVLLGTSSPSGRLPATWLYNWGVVPVLFPFGFGLSYTHFHTTDMRASPWDCRTRGDTGAGTAAEGRRRRQAAPRGAGGGGGEEELCFRVEVDVVNAGRRASGHAVLLSLRRSPQPPGAAPAAGGTRTGAGGGSGSGGGGARSDSGDAGGGGASGGAGGRVLAAAGVGGVAAAAASGGEPGAGRAHSGCRPCAEVLQQQQPWRAQRQQEQGQRDGPQDGEEESEEEEEGGEFAEWGGERYGHPDTLAAHTAAYHRIMADRLERPQGQQRPQAQQGQQGRGARGGSGARGSASAAGAGSHSDGGGGDELDGQVDGVDDDPPPVRELVAFGRLEDVSPGEVRTLVLQVRLPAAGLEVEVEAEAEVEAEAEVEAEEEQQAGRQGGGPRRRAHRRYVAHLQAGALCCPLPRPAASRR

>Cre14.g617600_CrDeg9.2

MFQLSPINCPSTALSVPSSRPRRHSVWRATGRHSRLNTSLLKTRSYGNDYSELQQLELASAGDAGGELPLNGGSPLIGALGSDDDAPEARTSAGPAAAPPIIKVTGELRRKAWALSSVLKVFVSKVDPNYAQPWQMCPQRTSTGSAFVLDTKKRQILTNSHVVSNATAVYVRRPGAARKFKAEVVCDGKVCDLALLTVRDDAFWAAELRGLEFVDVPELQSPIAVAGYPVGGDNISVTKGIVSRIALVRYSATARLLSIQIDAAINPGNSGGPAFADLEGGKVAGVAFSKNVSSSTDNIGYIIPYRVVRHFLEDAESHGTYRGVPSPGFFTQDLENPAQRAYLKMPEGVSGVMVVKTDPLSAAHGAIQKNDVALEVDGVPIADDGTVEFREDERLEFSAIIRAKHVGEQAHIKLLRDGQELCVSYELRAKDHLVPVLDAVDAVPSYLIVGGLVFVPLSSPFLEMVFGGGGGRRSRRADIPVPVLAALNQNKTRKGQQVVLLVQVLAHEINHGYRYSVVPCESFNGTRLHSLRHLAHLVDNCEQPFLNFGLEGGRLITLATADVRAAGPQILSTNAIASDRSPDMALPKPEDEDWDAAAADTVDENPVGGPTGGDSDGDG

>Cre01.g013300_CrDeg10

MPLQGRLLGASGPPAAHPSPASSLRTRNRAFCSAAPAAGAGWPLHSPSHNERGQPLAQLPHGAAAPAGRDWAPQPLSVTGGSSGVSAGAVGAASSSSSGGAAAAVGGWQAAPSLVPRVTTDDMLLSSDGAAAAASTSSAGGAGAAAAAAPSRRGRRGTAAAASGPGAAAAGGGDGGSSAAEDVAEPVVVHMSAAATATAAAGTMPAPRHTGVFQPSRSYSQHIKSDGELLAALAEAPLAYAEGMGSVLDSIIKIYTVHSRPNYTLPWQNHPKRESTGTGFVVHDRLILTNAHVVADATYVLVKRHGSGTKYRADVQAVGHDCDLALLSVTDEAFWSTPTSMLPLELGEVPELQQGVVVVGYPTGGDNTSVTSGVVSRVEVAQYAHAASHLMACQIDAAINPGNSGGPALQGDQVVGVAFQNLPGAENIGYIIPTPVVRHFLSEVRKYGSYQGYCSLGVLCQNLENPHLRSALGMGEGMTGVLVNTIQKTSNAAKVLKPGDVLLEFDGVRIANDGTVHLRQRERIYFSYLITLKPTAGTAKLKVLRDGQQLVFDMPVTPNDLLVPVHCYDRLPSYFMYAGLVFVPLTQPYLHEYGEDWMNTAPRRLYDKAMHGMMQKPRQQIVILSQVLVDDVNTGYQQFQTLQVLRVNGTEVLHLAHLKELVEGAADRFVRFELEDERVMVVERSLAVDANARIMERYRVPSSVSADIGANAAGAAASGASSGSTNGGASGELAAAAQQ

>Cre12.g548200_CrDeg15

MSSSLESVLVLISGEESADDPSQALSFVTLCAAGSVALGSGRAALLVPIATFSVPAAQRAASALLHGLNRDATSDAARWNFAWSIAATAEASPAEQGAYVLCLAQPLPDAPRAPPAPPAPVLHPDLLASVAPLLRSLRALLLPPGGAAASPCAACGAAQRSCCGACAAAAAGIAPPPGPGRLVTVVGSPFGCLAPFHFLNAHVNGAIACSFPPPHQHQHQPTGAMSGAGTAPAATTAAPPALASQQLQPQLQLQPALYALDAHVFPGMEGAPVTPLQLPLASLTGGLPAAAPPGASASSATIPSSQARSQGCGGTGCGGCGGCGGCGGCGGCGGCGGCGGAVAAAAAGWQRPVALLATPLCRQGDGVQVPLAVAWPHVEAALRELLQRLLAQVDGAGGGGGAGGGGPEAQQQQLLQLQPVSGSLARALAGGAWGSAAGGGGGGERRPWWDLTRMLPAGWGGEADAFGGGGGLGGGAGTLQWRRHHPHTSIIGSGSGNGSAAMTLDPCAGPPGADAALTTRAGGSAVAATNAAAAGISYSPALVSRQAVYAAGAYHHSPHTAQLQQHHHHHQPGPATNPAAGAPGPFPACAPACHGGACGGLGGAGGGIGAGGGGVGSELPPWALDAVVLLRCSGSWATGVLVESRSGLLVTTAHLFQRQHTSGGNGGGGGRSNGSGDSSNGDGSGRAGDGSDSSAWEHVVCWARVRCIRQGGYGGWYGGGGSASGATSHRWIRAKVVYVWNNHLDLAVLQLQSTHGGRWGVAPSPLETLGRAGGGADGGGGLAAAAITATARARDEDAESSRSSSGSSSNGSSSGSSNGNGSSSGASANSGRNLNVAGLFASAAGVAAELNAEVPAHPNTHAIHHHQHHHHLQQQQRHQQPAPPAMAALPLGSAGSYGSGTPVWAVGHSLVGPGAEWPPLVSFGNVARVLRGADGVPTMIIATTTTHAGGSGGALLDAAGRLVGLVTSNARHAGGATLPNMAFCIAAEELEPVLRWAAQRARLMQEGGQQEDEAAAAKLPLPPLPPLASLDGAQQHQHQPRQHQQVYEEQQEARGPREPLRQPAAAPWSRGGQPQSAAAGSAAAAAAAPCPSTAAAAPAARALSALDVRNEDASRIWRLQMPSFGPLHQAAARAAMTAGAVAGAAAAVAQSAPAPAAPGARRAPAAAADDGGVSATLRAIEVVRSWRSRL

>Cre07.g332050

MCSVLRGFGADLKQLDDSAVDALWKAGYRSEDLLQIATHKGLKDAGLLPAYVDYVLSLKGGGSRSAPDAGQPRDQQEDTGMQRVAASSKFTGRVPYDKVDPKTKQVSRLPSCVIVIAPKFAATFAHHRTWSEGQDVELEMYCSDANSMRAVPAKVFRIDTRVDFVILKFVGEELPAPAVAGGPQAGTGFYGHGLSLGGNTANVYHGIVARPAAGSRGHVALDKLSTKGDSGGGIFSAATGQLLGMMVGRDQEREISYMVPAAVLLAATAEDNFDPDPPPSGEWM

>Cre13.g579900

MPPRKKVKAEAAVVEDAKPGGRGGDTMSVTSGVVSRIEVTSYMHGSSELLGIQIDAAINSGNSGGPAFNDDGQCVGIAFQSLKHEDAENIGYIIPTPVIHHFLTDFERHGAYTGFPCLGVEWQKLENPDLRTVLKMKADQKGVLIRRVEPTSACSAVLRQNDVLMSFDGIAIANDGTVPFRSGERISFSYLVSNKYTDEEAELVVLQGRWVGLVFTAVTVPYLRSEYGKEYDFDAPVKLLEKMMHGMAQHKEEQVVVLSQVLAAEVNLGYEDICNTQVVSLNGGRITTLAELVARVDACDQPFLVLDLEYNQKVVLDTAKARAATSEILAMHCIATDRSPDLLGQQAAAAAAAAGAAAEAAGAGAAGAGPKEGEAEAEVKEEVAETNGAGAAAAGAAAGKAAAVGTRGGRRRLPA

>Cre03.g203730

MVGGIVACRVSTGPHASASLAGAGGEHAGARGAAWVCHATRPVRKPPLRMRAPPPQPEDLPRFDMPLLLGTFRSSTCNAVAVVHGGQRYLLAPSAAVAYGSQVRVYLPGREKPFPARVAHLAVDCELAALELIGSSSSSNGSNGSGSSSSSSSGGSAAAAAAVEEFWGALQPYQLADQGLPALQAAVGVVSYAEAQPQPSLSPGTVMRTEVITYPSALQRLLGLTVAVAISKEQLGSAVVDGRGQCLGVVFGRTVGSGRRKGGGAGSGAGSGGSGGGGSDSRWGQGRRRVGRRRGRRGQQEASALVVPVPVVAHFLEDLQKHGRYLGFPTLGIQWRRTESPALRRYTGMAPEQTGVAIVSINPTAALAAAGGQPLDVLAAVGDAAVGNDGTVAFRGGSESINISYHISQFQVGDTLDLTLLRRGAALTLPITLGVPGRLLPLHLAGAPPQWLVVSGLVLTVLSGPFLEGAFGRGWAVRAPVQLLREWHNHPASEDEQVVVVAECQDMGPGSATDGYERRGERASSQSPELGSGSGSGSSSGPPLDPSYITLELSSRLVMVLPLEVVVADTREMLGEYEVAHAVSEDLRTEYEGVMKARRQQLAAAGEKGAGAGEKAGKGKGKGRGGRSGKGR

>Cre38.g785300

MVSRTAVDKALRKKGLVEASELGVLLAFLQDQSDDQMSQWMIRDTDEPEIIAAFVRARAA

KDGDTTAHTDQGKGDQLDDNVVQQQLGATRKFTGQVVHDFTDPKTGKACQMPSCVTLIDTKYAATFAHRRTWTEGQDVELEICSGTDPNSMRAVKAKVFHINPELDFALLKLTDDEELQAPNIEPGPRDGMKFFGHGLSCPGGTSSNVCRGVITRAHMVGNRYIALDRISGKGDPGGGIFSAATGRLLGVLVGRDTDLDKSYMIQANVLLAATVEQHVCMMERLADDTGAGSSQAGSRRLMS
